# Allosteric regulation of glutamate dehydrogenase deamination activity

**DOI:** 10.1038/s41598-020-73743-4

**Published:** 2020-10-05

**Authors:** Soumen Bera, Mubasher Rashid, Alexander B. Medvinsky, Gui-Quan Sun, Bai-Lian Li, Claudia Acquisti, Adnan Sljoka, Amit Chakraborty

**Affiliations:** 1 School of Mathematics, Statistics and Computational Sciences, Central University of Rajasthan, Bandarsindri, Ajmer India; 2 Institute of Theoretical and Experimental Biophysics, Pushchino, Russia; 3 Department of Mathematics, North University of China, Shanxi, People’s Republic of China; 4 Complex Systems Research Center, Shanxi University, Shanxi, People’s Republic of China; 5 Department of Botany and Plant Sciences, University of California, Riverside, USA; 6 Institute for Theoretical Biology, Humboldt University, Berlin, Germany; 7 RIKEN Center for Advanced Intelligence Project, Tokyo, Japan; 8 Department of Chemistry, University of Toronto, Toronto, Canada

**Keywords:** Computational biophysics, Molecular biophysics, Single-molecule biophysics, Biophysics, Computational biology and bioinformatics

## Abstract

Glutamate dehydrogenase (GDH) is a key enzyme interlinking carbon and nitrogen metabolism. Recent discoveries of the GDH specific role in breast cancer, hyperinsulinism/hyperammonemia (HI/HA) syndrome, and neurodegenerative diseases have reinvigorated interest on GDH regulation, which remains poorly understood despite extensive and long standing studies. Notwithstanding the growing evidence of the complexity of allosteric network behind GDH regulation, identifications of allosteric factors and associated mechanisms are paramount to deepen our understanding of the complex dynamics that regulate GDH enzymatic activity. Combining structural analyses of cryo-electron microscopy data with molecular dynamic simulations, here we show that the cofactor NADH is a key player in the GDH regulation process. Our structural analysis indicates that, binding to the regulatory sites in proximity of the antenna region, NADH acts as a positive allosteric modulator by enhancing both the affinity of the inhibitor GTP binding and inhibition of GDH catalytic activity. We further show that the binding of GTP to the NADH-bound GDH activates a triangular allosteric network, interlinking the inhibitor with regulatory and catalytic sites. This allostery produces a local conformational rearrangement that triggers an anticlockwise rotational motion of interlinked alpha-helices with specific tilted helical extension. This structural transition is a fundamental switch in the GDH enzymatic activity. It introduces a torsional stress, and the associated rotational shift in the Rossmann fold closes the catalytic cleft with consequent inhibition of the deamination process. In silico mutagenesis examinations further underpin the molecular basis of HI/HA dominant mutations and consequent over-activity of GDH through alteration of this allosteric communication network. These results shed new light on GDH regulation and may lay new foundation in the design of allosteric agents.

## Introduction

Protein conformational dynamics are mainly regulated by changes in structural flexibilities. However the impact of the associated conformational rearrangements of amino acid residues on protein functions, ligand-bindings, and allosteric communications still remains poorly understood^1–3^. Comparative structural studies have shown the critical role of conformational rearrangements upon ligand binding to allosteric proteins^1,4–6^. These ongoing protein dynamics are highly complex, with several allosteric inhibitors potentially able to act at a large distance from the core catalytic domains and to inhibit the enzymatic activity in cooperation with a variety of cofactors^2,7^. Here we investigate the allosteric regulation of the mammalian glutamate dehydrogenase GDH and show how the Guanosine triphosphate (GTP) binding establishes allosteric communication with the regulatory coenzyme NADH which then synergistically inhibits the oxidative deamination activity of GDH by stabilizing its closed conformation.

GDH provides an essential link between carbon and nitrogen metabolisms^8^. Despite extensive studies for the last 40 years, its regulation remains elusive^9,10^. Recent discoveries of GDH specific role in breast cancer^11^, hyperinsulinism/hyperammonemia (HI/HA) syndrome^12^, and neurodegenerative diseases^13^ has reinvigorated interest on GDH regulation and given new momentum to the field. While the molecular structure of GDH is well resolved, the underlying dynamics of GDH regulatory system which involves several allosteric modulators remains poorly understood^14^. Important input has come from the genetic variation associated with the hyperinsulinism/hyperammonemia (HI/HA) syndrome in the human population. It was shown that most mutations are located near the GTP binding sites or in the adjacent antenna region^10,14,15^. Mutant GDH from patient lymphoblasts and in the mutant GDH expressed in *E. coli* showed almost complete unresponsiveness of GDH to GTP inhibition, normally reduced from 1.3- to tenfold compared to the wild type^16^. The molecular basis of the GTP inhibition and the associated GTP insensitivity are one of the key remaining open questions of the GDH regulatory system.

The role of the cofactor NADH in the regulation of GDH activity has emerged only very recently. It was previously observed that in absence of NADH, GTP binds only weakly to GDH. Single-particle cryo-electron microscopy (cryo-EM) studies have further revealed that the GTP binding affinity is increased in presence of NADH at the regulatory site, and GTP synergistically displaces the complex towards the closed conformational state^17^. However, the underlying mechanism explaining this effect has not been previously elucidated. A key problem is that these combinations are not amenable to crystallization due to the fleeting nature of the structures involved. Although there are several solved crystallographic structures of GDH complexes, these are either crystalized with cofactors and nucleotides or predominantly in closed conformation only. Using the available cryo-EM data of (i) the apo enzyme GDH without bound substrates (PDB id: 3jcz), (ii) the open form of NADH- and GTP bound GDH system (PDB id: 3jd3), and (iii) the closed form of NADH- and GTP bound GDH system (PDB id: 3jd4), here we have focused on describing and mapping out whole allosteric network behind GDH regulation. Our computational analysis and simulations have shown that the cofactor NADH is indeed a key player in the allosteric modulation, where it acts as positive allosteric modulator. The results presented in this study indicate that GTP activates a triangular allosteric network interlinking distant GTP binding sites, regulatory NADH, and catalytic sites. This network controls the Nucleotide-binding domain motion that eventually blocks the catalytic cleft in the GTP-induced inhibition dynamics. Moreover, using in silico dominant mutational analysis, we show that impeding the allostery network leads to GTP-insensitivity, resulting in GDH over-activity.

## Results and discussion

### A large conformational difference between open and closed GDH system

**Figure 1:**
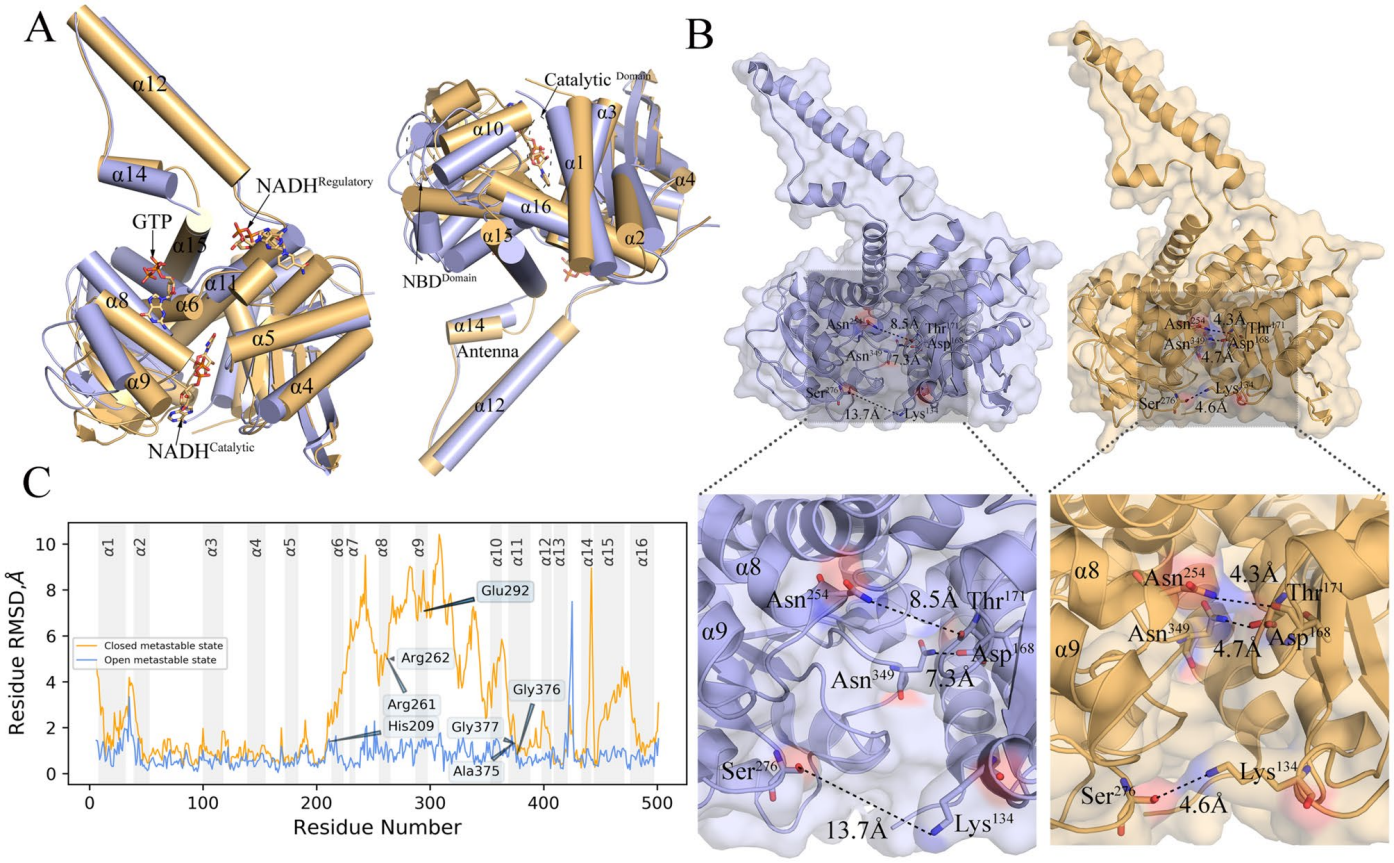
Conformational differences between the open-and closed states of GDH bound with NADH and GTP: Conformational differences between the open-and closed state of the GDH regulatory system, indicated by light blue and light orange color respectively. Two structures are superimposed using $C_{\alpha }$ atoms and α-helices. (**A**) 3D side view of opposite sides of α-helices and their transitional and rotational shifts. Most α-helical shifts are local at NDB domain in-between the GTP-binding sites and NADH-bound catalytic sites. (**B**) Local distance between surface residues within the GDH-catalytic cavity significantly reduces at the closed state relative to its open conformer. (**C**) The root means square deviations (RMSDs) of Cα atoms for each residue conveniently differentiate the two conformational states, with substantial conformational changes in the helices VI to XII and the antennae regions. Light blue color graph represents Cα RMSD between the open and apo-GDH form. Light orange color is used to show CαRMSD between closed and apo-GDH form [Software used: PyMOL Version 1.8.4.0 https://directory.fsf.org/wiki/PyMOL].

Cryo-EM GDH complex structure exists in open and closed conformations. Both forms have bound cofactor NADH and the inhibitor GTP having substantially different conformations. While the closed form is relatively well resolved and detailed structural knowledge has been accumulated during last two decades, the structure of the open form is not well understood. This is problematic because allosteric regulation is primarily driven by the inherent differences between the open and closed forms. Using a structural superimposition with RMSD fingerprint of individual residues, we have focused on the detail characterization of the differences between the open and closed structures. GDH has a homohexameric structure composed of a trimer of dimers, where each monomer consists of three identified domains^18^ (i) Catalytic domain, (ii) Nucleotide-binding domain (NBD), and (iii) the Antennae. Borgnia et al.^17^ solved the 3D cryo-EM structure of open and closed forms of GDH bound with both NADH and GTP. Within these structures, the catalytic domain is located near the dimer interface. Moreover, in both the open and closed states, adenosine and nicotinamide moieties of the cofactor NADH are present in nearly the same orientation. The Nucleotide-binding domain (NBD) contains the βαβ Rossmann fold that undergoes a large conformational change during the transition. We have focused on these particular domains and investigated in detail the local architecture of the catalytic cleft by measuring the distances of all the AA residues. We can clearly associate the closure of the catalytic cleft with changes in the distances between the following pairs of amino acid residues: Asn254-Thr171, Asn349-Asp168, and Ser276-Lys134. These pairwise distances are reduced at least twofold in the transition from the open to the closed form (Fig. 1B).

It was previously noted that within the catalytic cavity, both the adenosine and nicotinamide moieties of bound-NADH keep the same orientation irrespective of conformational forms^14,17^. Our detailed examination of this domain shows that eight hydrogen bonds keep the NADH bound in the catalytic pocket in both forms (Table S2). Comparison of the closed and open structures points to a critical shift in the positions of Ans349 and Ser276 residues involved in the formation of H-bonds. In the closed form Ans349 is the closest residue to NADH (2.45 Å), while in the open form Ser276 is the closest residue (2.47 Å) forming H-bond to the catalytic NADH. Furthermore, we find that the composition of AA residues that built-up the catalytic cleft differs significantly between the two forms. Asn374, Ala348, Ala326, Val255, Gly253, Gly251, and Thr215 contribute the most to make-up the catalytic cleft in the open form, while Asn374, Ala348, Ala326, Ala325, Gly256, Val255, Gly251, Thr215, Asp168, Pro167 are the primary adjacent residues in the closed form (Fig. S2). To determine whether the GDH-NADH interactions in the catalytic cleft change during the transition, we measure local interface area between NADH and GDH. We also noted that the interface area is reduced during transition from open to the closed form (Figs. S2, S3, Table S2).

At the regulatory sites in both structures which are located at the vicinity of “antennae”, the cofactor NADH is known to be bound as an inhibitor and then mutually facilitates inhibition of GDH deamination activity together with the GTP bound at a distant site. Borgnia et al. and Smith et al.^14,17^ observed that Adenine moiety of NADH has identical binding pocket as ADP, a potent activator of GDH and its conformations remain unaltered between the two conformers. However, nicotinamide portion of NADH has different conformations between open and closed state. It was further reported that His209, which undergoes large movement, plays critical role in NADH binding and stabilization when GDH system moves towards its closed form. Our distance calculations perfectly match this observation that the alpha-phosphate of NADH is ~ 4.4 Å away from His209 in the open form and is more than 10.5 Å away in the closed form. Furthermore, NADH binding engages other interactions and has altered adjacent residual compositions when open structure transits to closed form (Figs. 1B, S2, Table S3).

GTP binding site is located next to pivot helix at the base of antenna in the both the conformers. We find that the GTP-GDH local interface surface area increases (~ 48 Å^2^) while it moves towards the closed state (Fig. S3), indicating much closer interactions. These increase interactions we observe in the formation of three H-bonds between NADH and His209, Ser213, and Glu292 in the closed state (Table S4), which explain the stabilization of the GTP binding. Interestingly, we note that GTP-GDH interactions are strongly modulated by regulatory NADH with effects on the GTP binding affinity. For in silico affinity calculation, we use CSM-lig machine-learning method. In presence of NADH at regulatory sites, our calculation shows that GTP binding affinity is much higher (~ 2 kcal/mol) with more close interactions through 2 additional H-bonding. This close interaction with total 7 H-bonding explains the higher affinity of GTP. We further observe NADH-triggered local conformational rearrangement through inclusion and exclusion of H-bonding. Two closest H-bond interactions between the residue Arg261 and GTP (2.71 Å and 2.74 Å) in absence of regulatory NADH have completely been lost when NADH is bound at the regulatory site, while the AA residues Tyr262 (2.35 Å), His209 (2.36 Å), and Glu292 (2.72 Å) forms strong H-bonds to bound GTP in the closed conformational state in presence of regulatory NADH.

We have calculated the root means square deviations (RMSDs) of Cα atoms for each residue for determining interhelical differences between the two conformational states. This comparison shows that the helices I to V form a stable helical bundle core, with minimal structural changes. In contrast, the substantial conformational changes are observed in the helices VI to XII and in the antennae regions (Figs. 1A,C, S1). Substantial RMSD differences are noted in helices VI to XII. These differences are resulted in the rotational motions of the helices, which are indicated in our measurements of helical rotational angles with respect to the apo structure. We find that the highest rotational shift of the helix X (~ 20.1°) and XIV (~ 17.8°) with respect to corresponding apo structure (Fig. S4). The associated rotational directions show anti-clockwise movement of most helices VI to XII and the XIV in the closed state, indicating inclusion and exclusion of some specific interhelical interactions during the transition from open to closed state.

### Specific interhelical interactions are triggered by GTP binding

With the residue interaction networks (RINs) approach^19^, we examined interhelical interactions and identified specific residue-residue interactions between the helices by comparing the open and closed structures. RIN shows significant changes in the interactions among the helices VI–XII when the GDH transitions from open to closed form. To probe this further, we computationally calculated the differences of ^13^C, ^15^N, and ^1^H chemical shifts (∆δ) using a combined approach of machine learning and sequence alignment^20^. Chemical shifts were calculated for all residues laying on the helices with substantial rotations (Fig. S9). Although average differences in ^13^C ∆δ with respect to the apo structure for open and closed form (i.e., 0.80 and 0.75 p.p.m) are not significant, many residues of pivotal helix, α9, α10, and α11 show a large difference in ∆δ, indicating significant conformational rearrangement.

**Figure 2:**
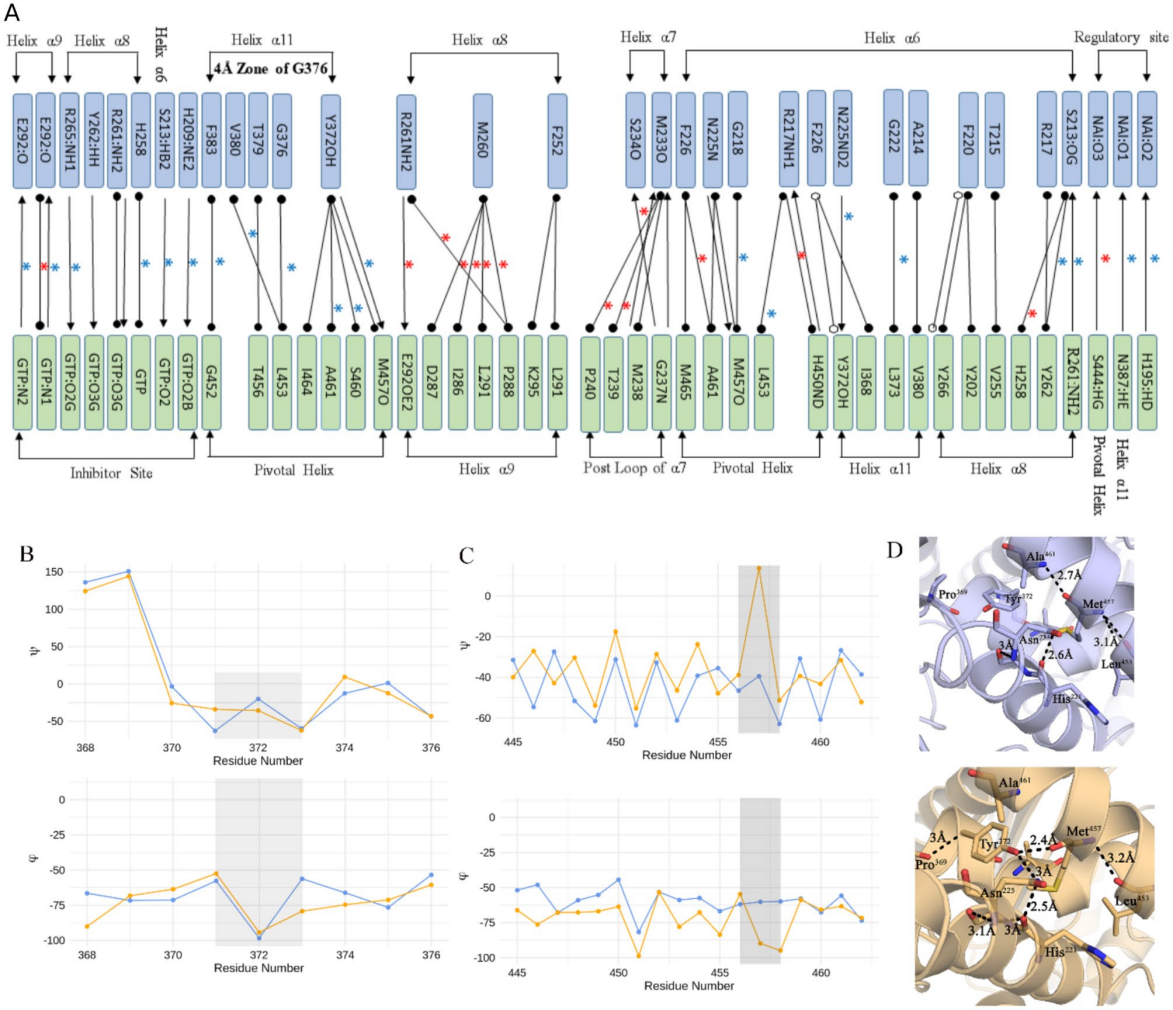
Conformational rearrangement and changes in interhelical interaction: Specific interhelical interactions that are changed due to conformational rearrangement of helices during GTP-triggered transition from open (blue) to closed (yellow) GDH state. (**A**) (*) represents absent interaction in the open form and (*) indicates absent in the closed form, while hydrogen-bond, van der Waals, and π–π interactions are notated as → ,  and  , respectively. (**B**,**C**) show the transitional changes in dihedral angles of residue Y372 of α11 and M457 of the pivotal helix. (**D**) Structural snapshots that represent the interhelical interactions between pivotal helix, α11 and α6 established in the closed form through H-bond interactions M457 (pivotal helix), Y372 (α11) and H221 (α6) [Software used: RING v2.0.1 https://old.protein.bio.unipd.it/ring/].

These calculations show that pivotal helix has retained the maximum number of interactions with other helices in both the forms. The most specific molecular interactions that are formed at the closed conformational state but lost in the open state are between the helix $\alpha 11$ and pivotal helix, including five Van Der Waals interactions and one strong H-bond interactions between Y372 and M457 (Fig. 2A–C). All these interactions occur within the 4.0 Å neighbourhood of GTP binding site. This reinforces that H-bond contact establishes a link between the pivotal helix, $\alpha 11$, and $\alpha 6$ via the Y372 that has H-bond contact with N225 of $\alpha 6$ (Fig. 2D). Loss of these H-bonds in the open conformation is reflected in ^15^N –^1^H plot of the chemical shifts, in which all the three residues show significant shift in the ^15^N–^1^H space (Fig. S9). Additionally, $\alpha 11$ residue N387 forms a H-bond with the regulatory NADH at closed state and thereby it provides a potential allosteric link between GTP binding site and NADH at regulatory site. Formation of these interactions underlies the large conformational differences observed between the open and closed form.

To examine the underlying dynamics of the conformational changes, we studied global dynamics of the GDH homohexamer emerged from the elastic network models (ENMs) of normal mode analysis. We used both the ENMs, Gaussian network model (GNM) and Anisotropic network model (ANM), available in DynOmics webserver^21^. GNM outputs effectively showed the multiple domain separations and per-residue contributions along the global modes. It also predicts the global hinge sites located between the sequence segments that undergo opposite direction movements along the slowest mode. ANM illustrated the collective dynamics along the global mode which is insensitive to the adoption of residue-specific force constants.

**Figure 3:**
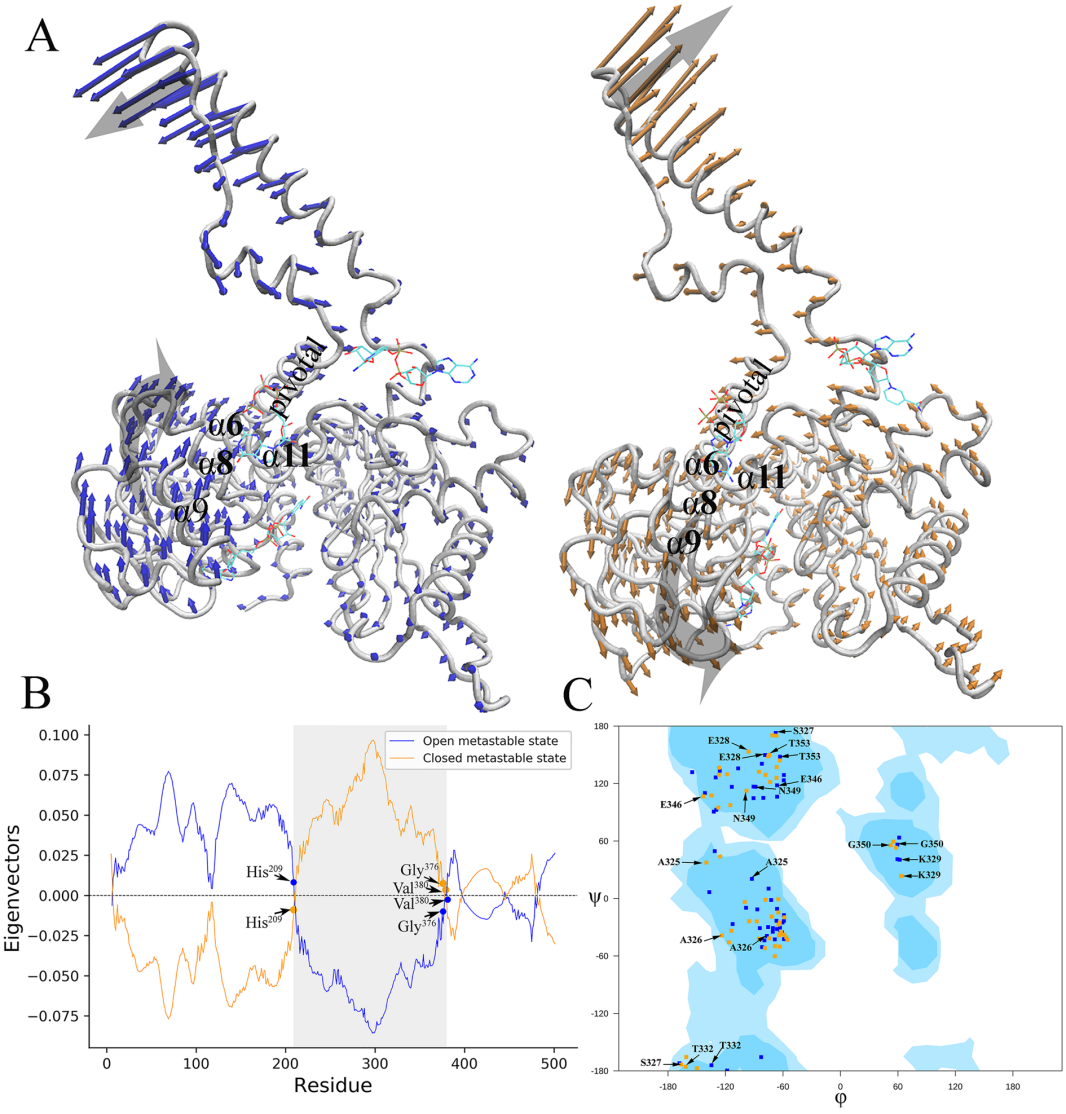
Relative directions and magnitudes of flexibility of open -and closed state of the GDH system. The low-frequency mode of a three-dimensional Elastic Network Model is calculated (**A**) Snapshots of opposite domain motions can be seen near to the equilibrium for the open (light blue) and closed (light orange) conformers. (**B**) The region around the AA residue 200–400 involving the helices VI to XII attained the picks of opposite motions and highest RMSD differences between the two states. (**C**) Ramachandran plot of the Rossmann fold AA residues that were superimposed on the contour plot generated with all phi-psi AA confirmations of 1000 frames generated by MD simulation. It indicates rotational shift of the residues as the open conformer moves to closed form [Software used: DynOmics Portal 1.0 https://dynomics.pitt.edu/].

ANM-driven collective motions along the second global mode with the RMSD 4 ${\AA}$ are compared between the open and closed GDH conformers. It showed that the size of the fluctuations and its spatial positions are different between these two forms, with the open form has larger structural flexibility particularly in the antenna domains compared to the closed form (Figs. S12, S13). However, intermediate Cα atoms excluding the antenna regions have reduced- and almost similar ANM-driven fluctuations. It, therefore, indicates the critical role of antennae for collective motion and for establishing inter-monomer communications by transferring GTP-triggered motions. Associated domain separations and motions along the global mode are evaluated using GNM. GNM-driven collective motions are characterized by frequency dispersion (reciprocal of the eigenvalue) and the degree of collectivity along the second global mode. A high degree of collectivity refers to a highly cooperative mode that engages a large portion of the structure, whereas a low collectivity means to normal mode that affect relatively small local regions. These calculations show that the open and closed GDH forms have the frequency dispersion of 33.528 and 32.037, and the degree of collectivity of 0.505 and 0.574 respectively along the second global mode. Mobility of residues along the mode also varies between these two forms. Differential contributions of residues along the domain motions are evaluated by separating the domains based on the direction (+/−) of their movement along GNM-driven second normal mode. Residues with same sign move together in the same direction and it is predicted to form a dynamically coupled regions. It also predicts the global hinge sites located between the sequences segments that undergo movements in opposite direction along the second slowest mode. Across all the six monomers, it shows domain separation of NBD and regulatory sites by the allosteric regions lie within residues ~ 200–400 (Fig. S13). At the onset of transitions, anti-clockwise helical motions are noted with the pivot helix rotated about 5.7° relative to the apo state. The nearly opposite domain motions along the normal mode of the two metastable states indicates some hinge-bending motions that are important for the transitions. It shows the region around the residue 200–400 involving the helices VI to XII, which attained the picks of the opposite motions and highest RMSD differences between the two states with the hinges around the residues His209, Gly376, and Val380 (Fig. 3A,B). To evaluate the NBD domain motion in isolation, we ran molecular dynamic simulation with the CHARMM36 force field and TIP3P water (see “Methodology” section) for 10 ns and generated 1000 structural snapshots at every 10 ps. With these 1000 frames, we produce a phi-psi contour plot on which the Rossmann fold AA residues are superimposed. This shows that most of the residues are rotated in the anti-clockwise direction within the predicted dynamic region (Fig. 3C).

Borgnia et al.^17^ described a large movement of His209 during the transition from open to closed state. In presence of GTP in the closed state, His209 swings away from the adenine moiety of NADH (~ 10.5 Å). In contrast, the distance between His209 and the alpha-phosphate of NADH is ∼ 4.4 Å in the open state, which is comparable with the corresponding distance in the potent activator ADP-bound conformation^18^. In addition to this result, we have identified another critical site near Gly 376 that show key role in the transition (Discussed in the next section).

### GTP-inhibition dynamics involve transition residues and changes in peptide-bond geometry

**Figure 4:**
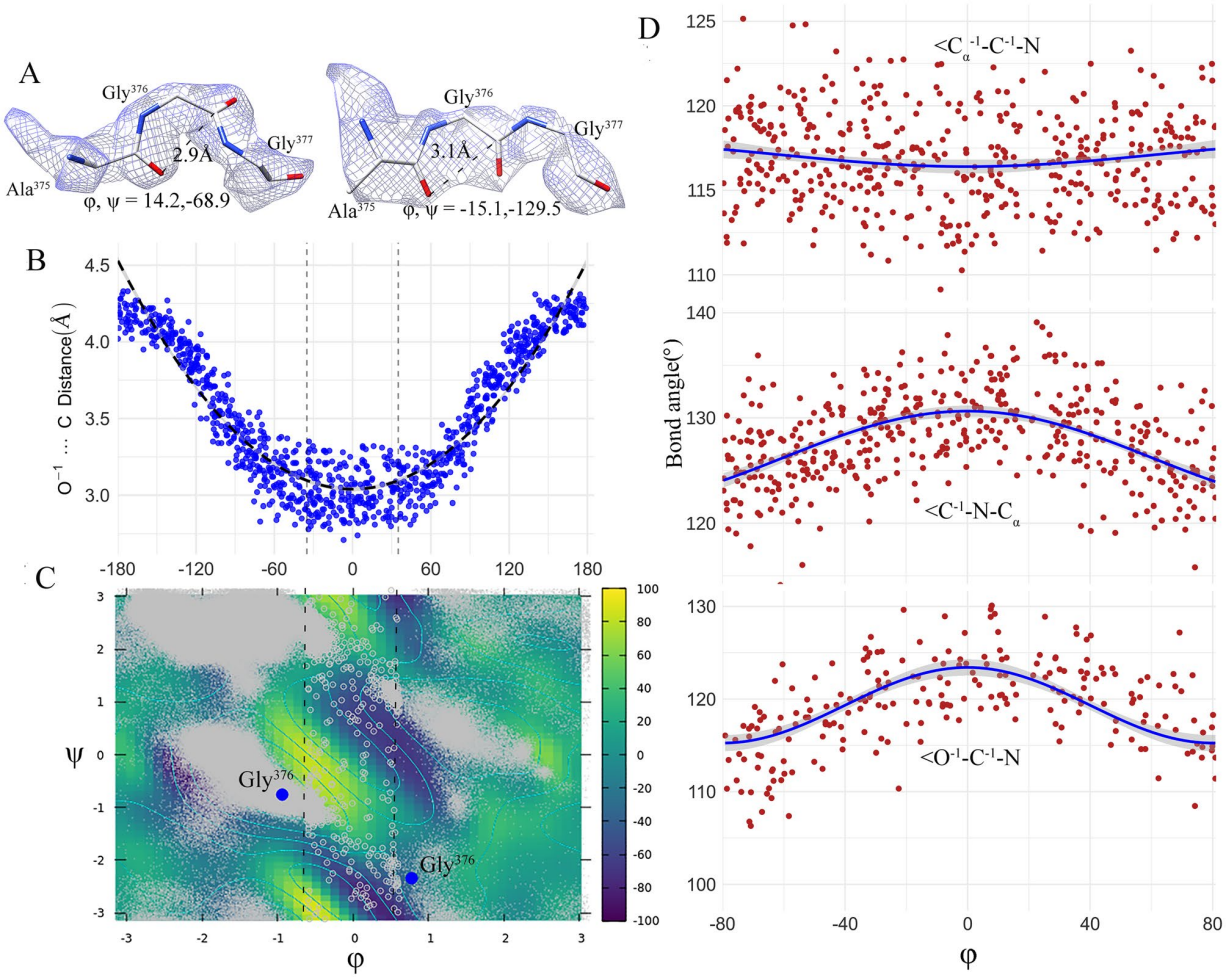
Transition residues for the conformational changes from GDH open to closed state: (**A**) cryo-EM map evidence for transient occurrences of Gly 376 in the transition zone ($- \; 35^{ \circ } \le \varphi \le + \; 35^{ \circ }$) which is a disallowed region in the Ramachandran phi-psi plot of protein backbone conformations. The tripeptide Ala375-Gly376-Gly377 conformations are the randomly chosen snapshot representations at 2.49 ns and 6.5 ns from the 1000 frames generated at every 10 ps by MD simulations of open conformer that ran for 10 ns using the force field CHARMM36. (**B**) Distortions of the distance O^−1^⋯C with respect to changesof φ along the MD trajectory. MD predicted data points (blue circle) are well-fitted with a quadratic function of φ: $y = \left( {5E - 05} \right)\varphi^{2} + 0.0001\varphi + 3.0397 \left( {R^{2} = 0.8813} \right)$. (**C**) A Ramachandran plot with energy contours for the tripeptide Ala375-Gly376-Gly377 calculated using adaptive biasing force methods. It used residues from 1000 frames generated by MD simulations. Small grey dots refer to non-transient residues sitting in the allowable regions of Ramachnadran plot and grey circle refers to transient residues located at the transition zone. It further showed that Gly 376 (blue solid circle) crossed the high energy barrier near to $\varphi \approx 0^{ \circ }$ through the low energy passes at $\Psi \approx - \;90^{ \circ }$. (**D**) Systematic distortions of the bond-angles with respect to φ. MD predicted data points are smoothen by a cosine function: $y = I\left( {\cos \left( {\varphi *pi/120} \right)} \right)$ [Software used: PLUMED version 2.5 https://www.plumed.org].

To determine and characterize the local effects of Gly376 conformational changes, we run the well-tempered metadynamic simulation in PLUMED v2.2.3^22^, using dihedral angles as collective variables. This simulation predicts the angular and distance distortions in standard peptide-bond geometry for the tripeptide Ala375-Gly376-Gly377 (details about peptide-bond geometry Fig. S6). Metadynamics-based free energy surface (FES) calculation for this tripeptide shows three energy picks near = φ0° that were classically described as disallowed regions in the Ramachandran phi-psi plot of protein backbone conformations. In between the picks, two low energy passes are found (Fig. 4C). The Gly 376 passes the high-energy transition zone (i.e., $- \;35^{ \circ } \le \varphi \le + \;35^{ \circ }$) through this low-energy passes with $\Psi \approx - \;90{^\circ }$. The Gly 376 apo-conformation ($\varphi = 47.1{^\circ } , \Psi = - \;109.3{^\circ }$) transits to the conformation ($\varphi = - \;60.5{^\circ } , \Psi = - \;43.2{^\circ }$) at the closed state via the open state conformation ($\varphi = - \;53.4{^\circ } , \Psi = - \;43.6{^\circ }$). Along the transition trajectory computed using the molecular mechanics force field CHARMM36, Gly 376 has repeated highest number of time among all the 14 different AA residues trapped in the transition zone, indicating the key role of Gly 376 by the distortion of backbone bond-angles of the tripeptide (Table S7). We have visually checked the conformational occurrences of many transition residues against its electron density map, and found that they are reliably defined (Figs. 4A, S7).

Distortions of the distance O^−1^⋯C and tripeptide backbone bond angles show the generic transition properties and it depends on the torsional angle φ along the trajectory. Distortions are φ-dependent and can be predicted by a quadratic function of φ with the $R^{2} = 0.881$ (Fig. 4B). Near $\varphi = 0{^\circ }$, the distance is relatively flat and down from the values not in the transition zone. The bond angles ∠C^−1^–N–C_α_, ∠O^−1^–C^−1^–N, and ∠C_α_^−1^–C^−1^–N show systematic variations, with average distortions of roughly ~ 2°, ~ 4°_,_ and ~ 2° respectively from their standard values. The bond angle ∠C^−1^–N–C_α_ has the maximum distortion of roughly 6° in the transition zone near to = φ22° (Fig. 4D and the details of other five angle is shown in Fig. S8. These backbone bond angles are expressed as a function of φ along with cosine function that fit to the data. This smooth curve for both the angles ∠C^−1^–N–C_α_ and ∠O^−1^–C^−1^–N display “humped”-type distortions with the hump near to = φ0°. However, the fitted curve for ∠C_α_^−1^–C^−1^–N remains relatively flat in the transition zone $- \;35{^\circ } \le \varphi \le + \;35{^\circ }$. This analysis therefore shows the key role of Gly376 to cross the energy barriers between the open and closed metastates.

### GTP-binding forms an allosteric nexus

GTP-binding triggers an allosteric response connecting the three distant regions of GTP binding, NADH regulatory, and catalytic sites and thereby forming an allosteric nexus (Fig. 6A). To ensure that allosteric sites are in sufficient distance from the catalytic site, we have calculated distances between the nearest atoms as well as Cα atoms of nearest H-bond forming residues of the allosteric and catalytic sites. We find that distances from GTP to NADH_reg_, NADH_cat_ sites are more than ~ 7.0 and 11.0 Å respectively. Mutation allosteric sites GLY376ASP, ARG217CYS, SER441LEU have maintained the distance > 10.0 Å from the catalytic site. These distances are further increased in the closed GDH form where allosteric communications become stronger than the open form (Table S9, S10).

We have evaluated the associated allosteric network using graph theoretical method FIRST^23^ which is based on pebble game algorithm and techniques in rigidity theory. In the rigidity-based allosteric communications model, a site is allosteric if perturbation of its rigidity/flexibility results in a quantifiable change in rigidity (specifically conformational degrees of freedom (DOF)) in the active site. When there is a change in DOF in allosteric site, rigidity-based allostery algorithm mathematically certifies and quantifies the corresponding change in DOF in the active site. We also point out that allostery can occur even in cases when there is a minimal conformational change that allosterically propagates to the active site. In allosteric effects it is not necessary to have major backbone coupled movements between active and allosteric site, instead changes in the side chains motion can also contribute to allosteric response. Backbone and/or side chain allosteric coupling can result in modifications in hydrogen bond network through allosteric pathways and transmit and modulate allosteric responses and conformational changes in the active site.

**Figure 5:**
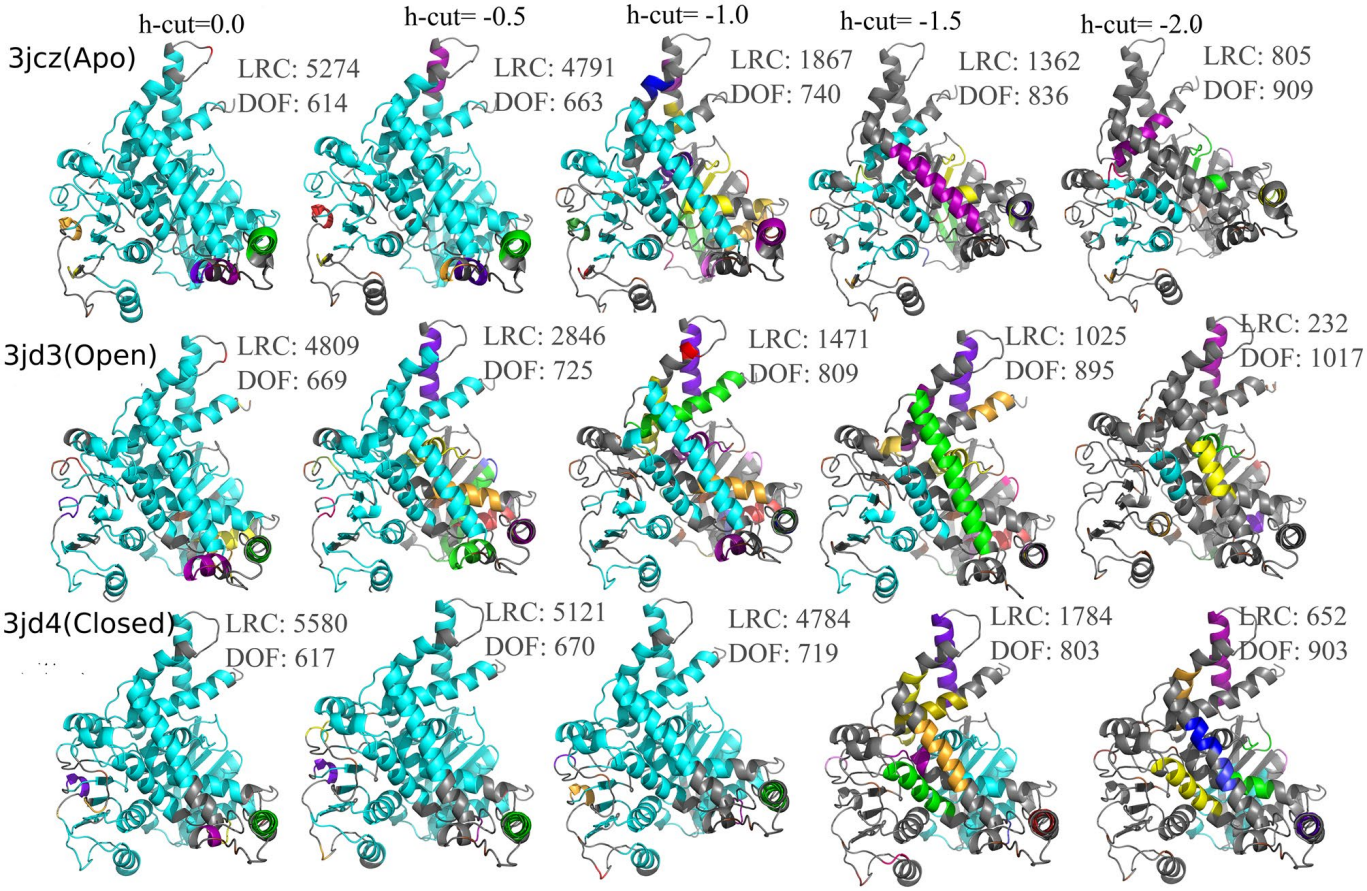
Rigid cluster decomposition of the GDH system with various hydrogen-bond energy cut-offs. The largest rigid cluster (LRC) is represented by cyan color and its size is progressively reduced as H-bond energy cut-off decreases from 0 to − 2.0 kcal/mol. H-bond cut-off − 0.5 kcal/mol is considered as acceptable cut-off that indicated by the H-bond dilution plot (Fig. S2) [Software used: ProFlex version 5.2 https://github.com/psa-lab/proflex].

FIRST efficiently decomposes the protein into rigid clusters and connecting flexible regions. Activation and functioning of allosteric network are indicated by the formation of a largest rigid cluster (LRC) that encapsulates the AA residues from a small neighborhood of these three distant sites (Fig. 5). Rigid cluster decomposition of apo, open and closed GDH structures with the acceptable H-bond energy cutoff of − 0.5 kcal/mol (Fig. S5) shows that GDH closed conformer has the largest LRC, roughly about ~ 1.8 times bigger than the LRC in the open form, with lowest total independent degree of freedoms (DOF). As expected GTP-bound open conformer has the smallest LRC and highest DOFs among all the three structures, referring to formation and activation of allosteric network through conformational rearrangements of LRC residues on the onset of GTP binding. Moreover, we note that the LRC of GDH closed conformer includes approximately twofold more helical structures than its open form, making it much less flexible and potential for intense allosteric communication. Consequently, total number of rigid clusters has significantly reduced when it transformed from open to closed form. In particular, α11 that play important role in the GTP-inhibition dynamics has contributed 84% for LRC formation in the closed form in contrast to its 33% inclusion in the open form (Table S6).

**Figure 6:**
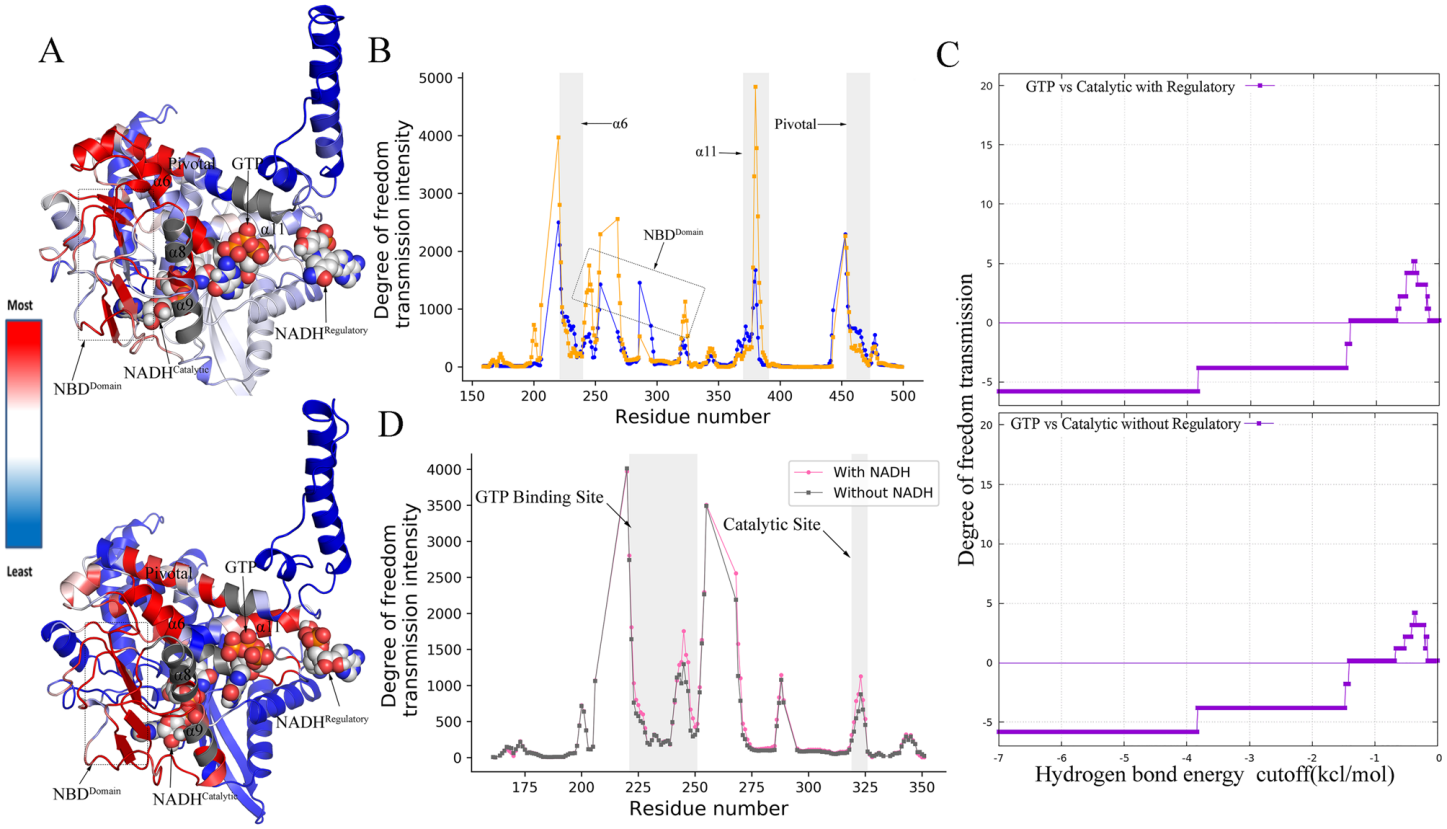
Long range rigidity-based allosteric communications: (**A**) Perturbing rigidity of pocket defined by atoms within 3.0 Å neighborhood of GTP ligand results in allosteric modification of rigidity and conformational degrees of freedom in the catalytic and regulatory sites. Highest degree of freedom transmission intensity (i.e. strongest allosteric effect) is represented by red color and lowest intensity represented by blue. (**B**) Allosteric hot spots are identified by grey sheds; allosteric intensity at the hot spots is much higher in the closed form (light orange) relative to open conformer (blue). (**C**) Total area under the allosteric curve is higher in presence of regulatory NADH. (**D**) Synergistic allosteric effects of GTP and regulatory NADH at the catalytic site are significantly higher relative to the GDH without regulatory NADH [Software used: ProFlex version 5.2 https://github.com/psa-lab/proflex and PyMOL Version 1.8.4.0].

Binding of GTP imposes local constrains by introducing two strong H-bonds with Arg 261. Furthermore, this local constraint has become more stringent in presence of NADH cofactor at the regulatory site which includes the formation of two more H-bonds with Tyr 262 and His 209. This GTP-induced perturbation in the local conformational rigidity within the 3.0 Å neighbourhood of GTP is transferred to other remote sites allosterically, resulting in a large NBD motion that closes catalytic cleft. To examine this allosteric effect quantitatively, we apply the rigidity transmission allostery algorithm (RTA)^7^ that predicts and quantifies the potential allosteric communications between the GTP binding site and the other remote sites. Residues are labeled as allosteric hotspots based on the intensity of transmission of degrees of freedom (DOF). Initially, both the GTP-bound GDH open and closed conformers were decomposed into rigid clusters and flexible regions using the program Floppy Inclusion and Rigid Substructure Topography (FIRST) and then RTA algorithm are applied to calculate if DOF propagate through protein structure as a result of rigidifying the GTP binding pocket. The RTA algorithm evaluates if perturbation of rigidity in GTP-binding neighborhood has caused changes in rigidity and conformational degrees of freedom in the catalytic and regulatory sites (i.e. allosteric transmission). Overall, RTA analysis shows that average intensity of transmission of DOFs is significantly higher in the closed state compared to open GDH conformer (Fig. 6B). Excluding the antenna domain that holds a large intrinsic motion due to its long protruding geometry and slight conformational changes in its immediate vicinity, residues 379–385 residing on the helix α11 are displayed to be allosteric hot spots, indicting the transfer of local rigidity to regulatory NADH sites. Simultaneously, residues 220–223 resided on α6 are allosteric hot spots. RTA analysis therefore shows allosteric communication between the binding sites of GTP, catalytic NADH, and regulatory NADH. This triangular allostery triggered by GTP binding synergistically affects the GDH catalytic activity and eventually blocks the catalytic cleft.

To probe the allosteric role of regulatory NADH, we further applied the RTA algorithm on the GTP-bound GDH system in presence and absence of regulatory NADH. Perturbation effects of rigidity within 3.0 Å neighbourhood of GTP on the catalytic sites are calculated based on allosteric intensity for both cases and then area under the allosteric (degree of freedom transmission) curve (AUCs)^24^ are calculated numerically that indicate the overall strength of allosteric intensity in presence and absence of regulatory NADH (Fig. 6C,D). AUC in presence of regulatory NADH is significantly higher than the AUC in absence of regulatory NADH, implying the specific role of regulatory NADH as positive allosteric regulator. Moreover, synergistic effects of GTP and NADH are reflected in the DOFs transmission intensity particularly at catalytic region which are much higher compared to the DOF intensity transmitted without regulatory NADH. In parallel to FIRST and RTA, we further used *AlloSigMA*^25–28^ to re-evaluate the predictions of allosteric communications between allosteric sites GTP, NADH_reg_, and the catalytic sites triggered by GTP-binding. It estimates per-residue allosteric free energies resulting from GTP and NADH_reg_ binding. Positive sign shows the effects of local destabililization, whereas negative sign indicates effects of local stabilization. While comparing the open form with the closed one, it shows stronger destabilizing effects in the potential allosteric sites (residues 200–400 excluding the antennae) with the ∆g (kcal/mol) ranges between 0.0 and 0.4 which is largely reduced in the closed form (Figs. S10, S11).

### Mutation of allosteric hot spot residues alter the allosteric communications

In order to examine how the triangular allosteric network responds to the mutations of key residues, we have prepared three GDH mutants in silico using Chimera rotamers tools^29^ GDH_Ser441Leu_, GDH_Arg217Cys_, and GDH_Gly376Asp_. As reported by Stanley^15^, GDH_Ser441Leu_ mutant is associated with the HI/HA syndrome and is the result of a substitution from Serine TCG to Leucine TTG codon in the *GLUD1* gene that encode the enzyme GDH. Recent study further noted that Ser441Leu is the most frequent sporadic mutation among the HI/HA patients^12^ (Table S5). This mutant shows over-activity of GDH caused by extreme insensitivity to GTP inhibition; however, the molecular explanations of this insensitivity remain unknown. We have recalculated the distance requirement of allosteric mutation sites from the catalytic sites. All the three primary mutation sites (Tables S9, S10) have the distance > 10.0 Å in both the forms. Moreover, the dominant mutation SER441LEU in the HI/HA syndrome is located at > 25.0 Å from the catalytic site.

**Figure 7:**
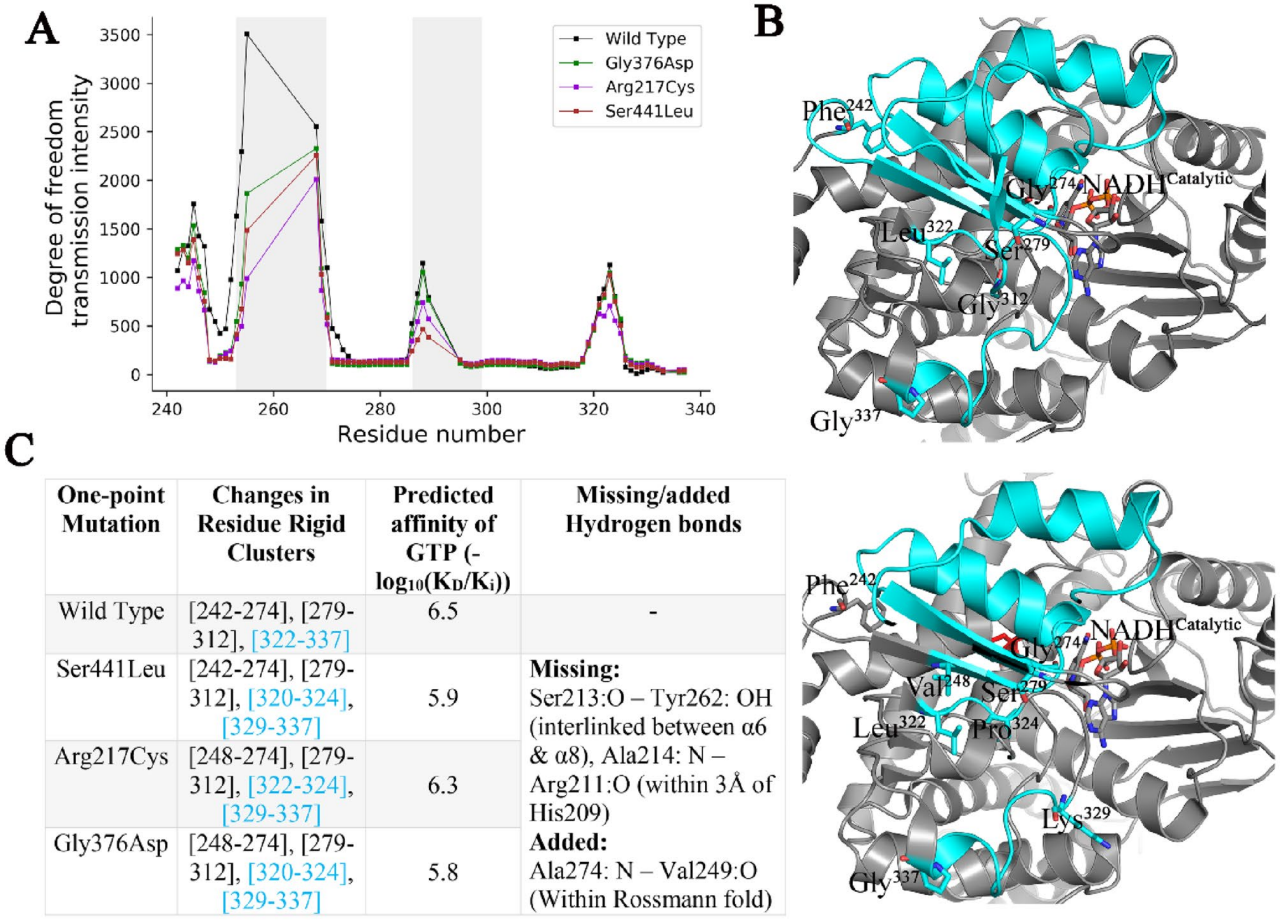
Point-mutations of transient residues: Gly376Asp, show the similar effects with that of the reported mutations Ser441Leu and Arg217Cys in HI/HA syndrome, in which GDH remains insensitive to GTP inhibition. (**A**) All the stated mutation has undermined the allosteric communications by reducing transmission intensity of the DOFs in-between the rigid allosteric hotspot regions, (**B**) an allosteric rigid cluster (cyan color) is broken into two parts after the mutations, (**C**) changes in the rigidity clusters and GTP-binding affinity under the one-point HI/HA mutations and the mutation Gly376Asp [Software used: UCSF Chimera-1.13.1 https://www.cgl.ucsf.edu/chimera/olddownload.html and ProFlex version 5.2].

To probe the effects of mutants on allosteric network, we repeated the RTA allosteric analysis of the GDH mutants. The RTA calculations with Ser441Leu mutation has undermined the allosteric communications by reducing transmission intensity of the DOFs in-between the rigid allosteric hotspot regions (Fig. 7A). Furthermore, mutation Ser441Leu destabilized rigid cluster [resi.322–resi.337] in the closed conformation where rigid cluster broke off into two parts. In particular, this has caused a loss of two H-bonds between Ser213.O-Tyr262.OH and Ala214.N-Arg211.O (Fig. 7B,C). This change in the rigidity disconnects the catalytic region from the GTP-binding sites, resulting in reduced motion of the Rossmann fold that fails to block the catalytic cleft as it observed in the closed type GDH conformation.

In parallel to Ser441Leu mutation located in the vicinity of the GDH antenna, GDH_Arg217Cys_ mutation is also associated with HI/HA and leads to insensitivity to GTP inhibition^15^. Although Arg 217 is located in the GTP-binding domain, GDH_Arg217Cys_ mutant shows similar effects on the allosteric network, including the inclusion and exclusion of the same H-bonds (Fig. 7B,C). Notably, binding affinity of GTP remains unaltered in both the mutations. As the residue Gly 376—a key transient residue responsible for the transition from open to closed conformation, Gly376 is substituted by Asp following the list of reported HI/HA associated mutations of GLY. Interestingly, it is noted that the mutant GDH_Gly376Asp_ mimics the similar effects on the allosteric network. Moreover, we performed the metadynamics on the GDH_Gly376Asp_ mutant and then recalculated the free energy surface (FES) for the tripeptide Ala375-Asp376-Gly377. The predictions show the loss of the two low energy passes as noted for the Gly376, which has prevented the crossing of the energy barriers by Asp 376 (Fig. S7D, Table S8). Therefore, this in silico mutational analysis reinforces the key role of the transient residue Gly 376 for maintaining the GTP-induced inhibition dynamics.

## Conclusion

The GDH regulatory systems involve a variety of protein effectors and many small allosteric modulators that build up a complex allosteric network. Recent discovery of HI/HA syndrome show GDH dysregulation through insensitivity to its usual inhibitor GTP and thereby over-activity of GDH^12,15^. Here we have illustrated an allosteric regulation of GDH by GTP and provide a molecular explanation for the dominant HI/HA mutations Ser441Leu and Arg217Cys that causes GTP-insensitivity. The details of regulatory insights of GDH system are also important to understand the fundamental link between carbon and nitrogen metabolism, as the GDH catalyzes the deamination reactions that supplies the alpha-ketoglutarate as inputs to TCA cycle. In this study we have shown how a purine nucleoside triphosphate, Guanosine triphosphate (GTP), binding establishes rigidity-based allosteric communication with the regulatory coenzyme NADH and then synergistically inhibits the oxidative deamination activity of mammalian GDH by stabilizing its closed conformation. This work, therefore, contributes to better understanding of the complex link between protein flexibility, allostery and protein function.

**Figure 8:**
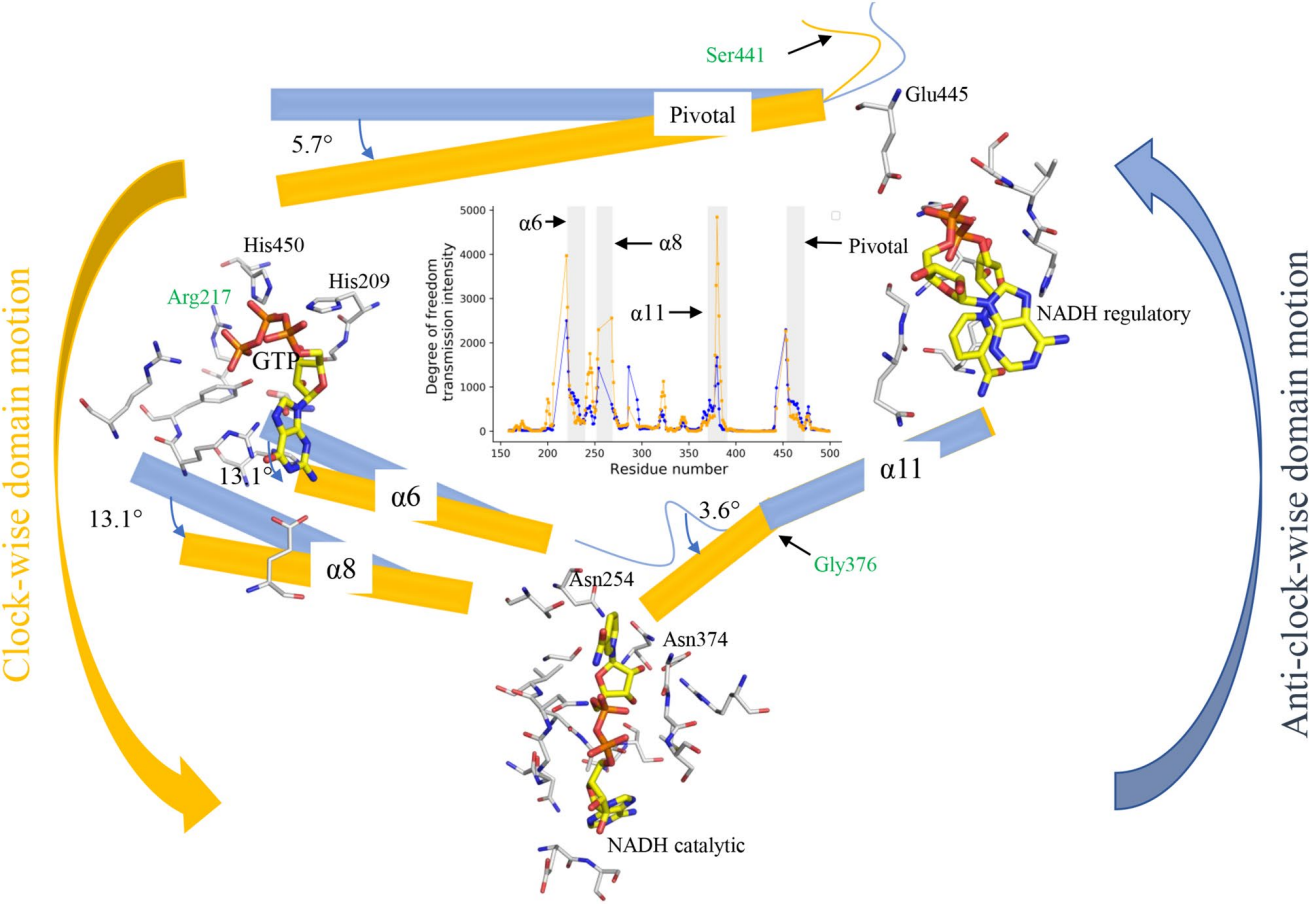
GTP-induced GDH inhibition dynamics. Allosterically interacting helices rotate anti-clockwise, in which the transient critical residue Gly 376 has a large conformational change (yellow represents the *closed* and the blue for the *open* GDH systems). It generates a torsional force, resulting in tilted extension of the helix α11 giving a rotational motion to the Rossmann fold that block the GDH catalytic cleft. The dominant HI/HA mutations Ser441Leu and Arg217Cys break this allosteric network, causing GTP-insensitivity and thereby over-activity of GDH. GDH_Gly376Asp_ mutant shows similar effect, inferring the key role of Gly 376 for the inhibition of GDH activity by GTP.

In particular, we show that GTP-binding triggers interhelical interactions and subsequent anticlockwise motion of nucleotide-binding domain (NBD) involving the Rossmann folds that eventually close the catalytic cleft. This NBD motion is controlled through a triangular allosteric network linking the GTP binding, regulatory NADH, and catalytic sites. It is further noted that the residue Gly 376, a critical residue located at the N-terminal of the alpha-helix 11, play an important role through local geometric changes in the tripeptide bond. These alterations result in a tilted extension of the helix 11 towards the NBD and generated a torsional force that provides the anti-clockwise motion to the Rossmann fold (Fig. 8). In silico mutational comparative analysis shows the similar allosteric effects of Gly376Asp mutation, as observed for the dominant HI/HA mutations. Moreover, the analysis illustrates the role of the cofactor NADH as a positive allosteric modulator (PAM), increasing the allosteric effect by increase in intensity of transmission of the degree of freedoms (DOFs) through the allosteric network. In addition, regulatory NADH enhances GTP binding affinity, with synergistic inhibitory effects on the GDH catalytic domain.

## Methods

### Systems construction for MD-simulation

The open form of NADH- and GTP bound GDH system (PDB id: 3jd3) cryo-EM structure was chosen and ligands, water molecules, other solvent and unwanted heterogens were solved and removed from structure by PDBFixer^30^ at pH 6.8. The fix structure was collected without adding missing hydrogen atoms. All hydrogen atoms were placed with the GROMACS tool pdb2gmx. All three separate ligands GTP and both regulatory and catalytic NADH were placed into Avogadro program^31^ for adding hydrogen and producing .mol2 file. We have used standard sort_mol2_bonds.pl perl program code for fixing the bond listing and other small error like those atoms were assigned different residue name and number. Topology and parameter files were constructed from final corrected structure with the help of CGenFF^32^ web server. Finally, Gromacs formatted topology was constructed using the cgenff_charmm2gmx.py script with CHARMM36 force field^33^ and added with protein structure topology file to generate protein–ligand complex.

### MD simulation and parameters

Molecular Dynamics simulations were organized and carried out with GROMACS v5.1.4 simulation package^34^ of version 5.1.4. Protein–ligand complex was solvated in a rhombic dodecahedral unit cell consisting of 41,638 water molecules, 90 Na^+^ and 82 Cl^−^. The complex was modeled with CHARMM36 force field and TIP3P water. All the MD simulation steps include energy minimization; equilibrations and production were performed by 2 fs time steps. The system was subjected to maximum 50,000 steps of steepest decent minimization or up to the maximum force dropped less than 1000 kJ/mol/nm to remove unfavorable energy contacts. Equilibration were carried out by two successive blocks of simulation with 0.1 ns each in the NVT and NPT statistical mechanical assembles where all bonds including heavy atoms-h bonds were constructed. The production simulation was performed with randomized initial velocities of all atoms total up to 10 ns at a temperature of 300 K. These simulations was carried out in the NPT assembles by heating to 300 K with the Nosé-Hoover thermostat^35^. Pressure (1 bar) was maintained using the Parrinello–Rahman barostat^36^ with coupling constant of 2 ps. Coulomb long-range electrostatics interactions were evaluated using the smooth particle-mesh Ewald method^37^ with cubic interpolation of order 4 and a Fourier grid spacing of 0.16 nm. Bonds to hydrogen atoms were constrained with LINCS^38^.

### Metadynamics

Well-tempered metadynamics simulation^39^ was performed on Gly376 by GROMACS patched with PLUMED v2.2.3^22^, using as collective variable (CV) the backbone dihedral angle phi and psi. The stride of Gaussian deposition was updated over every 500-time steps while the height of the Gaussian potentials was set in 1.2 kJoule/mol. The width of the Gaussian potentials for each CV was specified to 0.35 rad at temperature 300 K.

### Curating the MD trajectory data for high-energy pass and protein geometry

From the MD trajectory data, 1000 structural snapshots were created over every 10 ps time step. On the basis of these snapshots, all the observation having their φ torsion angles in the high energy pass region (− 35 < φ <  + 35), were manually curated. Reliability of the structural conformation was tested on the basis of visual assessment of the fit to their Electron Microscopy (EM) map with Chimera v1.3 package Fit in Map29. Reliable structures were separated whose φ torsion angle belongs to high energy pass region. Unreliable or close to unreliable residues were also included in this range to present of alternative conformations. All this curated observed residue in this range extracted from MD trajectory data by writing a custom script in GROMACS and GNU plot. Some specific geometric details were also calculated for all residues from each protein snapshot, excluding those residues not having two residues of both sides of it. The geometrical quantities include backbone torsion angles, bond angle and the O^−1^⋯C distance.

### GNM and ANM analyses for protein dynamics

Anisotropic network model (ANM)^40^ and Gaussian network model (GNM)^41^ analysis was done using DynOmics web server (https://dynomics.pitt.edu/)^21^ and Bio3D version 2.3.0. of R package^42^. Relative mode of motion was identified along the principle mode using GNM. Block of residues were divided into separated domains based on the direction (+/−) of their moment in the slowest modes. The sign (+/−) of the elements (residues) based on the selected mode eigenvector. Same sign indicates the structural regions that have correlated (positive) motion and opposite sign linked with anticorrelated (negative) motion. Overlap analysis was carried out using Bio3D to identify which modes contribute a given conformational change. Structure visualization, analysis and animation were performed in the NMWiz (Normal Mode Wizard, Version 1.0) tools of VMD^43^.

### Rigidity-based allosteric computations

The three-way allosteric transmission from NADH regulatory site to catalytic site through GTP binding site, is observed using RTA algorithm, a computational approach based on rigidity theory^44^ and an extension of FIRST method^23^. The RTA algorithm was first initiated in 2012^45^ and further developed and discussed by Whiteley et al.^46^. This algorithm extends the pebble game algorithm to predict whether local perturbation of rigidity at one site of the protein, transmit across the structure to change the rigidity of the second distant site. FIRST generates a constraints network from the coordinates of the protein structure in terms of nodes (atoms) and edges (covalent bonds, hydrogen bonds and hydrophobic interactions). Hydrogen bonds were ranked according to their energy strength using Mayo potential^23^, whereas the value of energy strength was selected in such way that the bonds strength below this cut off were ignored. First then applies the rigorous mathematical theory of rigid and flexible molecular structure and pebble game algorithm^47^ calculates the degree of freedom of motion to rapidly decompose a protein into rigid clusters and flexible region. We applied RTA analysis determines whether perturbation of rigidity and conformational degrees of freedom at NADH regulatory site could propagate through the protein structure (Pivotal helix, α11 and Rosenman fold) inducing the quantifiable changes in rigidity and available number of degree of freedom at a second distant site (GTP binding region), hence result in allosteric transmission. The number of conformational degree of freedom at the GTP binding region was calculated before and after a sequential perturbation of rigidity of all residues at the regulatory NADH binding region. To understand the role of regulatory NADH, the changes of number of conformation degree of freedom also measured after removing the regulatory NADH from the structure.

To prepare for rigidity and allostery analysis, missing hydrogen atoms were added to Cryo-EM structure using WHAT IF web server (https://swift.cmbi.ru.nl/servers/html/htopo.html). We sequentially perturbed the rigidity of a window of the three-consecutive residue starting from N-terminus, to calculate maximum degrees of freedom that can be transmitted to the GTP binding region. Perturbation of rigidity of a site means insertion of additional constraints (edges) (removal of degree of freedom) up to its rigidification. Transmission of degrees of freedom indicates a subsequent change in degrees of freedom at GTP binding region and the presence of rigidity-based allostery. We define binding site as all atoms that belong to 3 Å neighborhood of GTP and NADH. The RTA algorithm computes the transmission of degree of freedom between two sites, where we define NADH regulatory site as site A and GTP binding site as site B. Details in how transmission of degrees of freedom is performed has been previously described^7,48^. This calculation was repeated by successively omitting week energy constraints upon increments of 0.01 kcal/mol. Positive degree of freedom transmission indicates the sites A and B are involved in rigidity based allosteric transmission. The intensity of allosteric transmission for a given residue was calculated from the average degree of freedom intensity of three consecutive windows containing that residue. Transmission of allosteric intensity considers the number of degree of freedom that could be transmitted and persistence of the transmission as a function of energy strength.

### Area under curve

$${\text{AUC }} = \sum \left( {x_{i} - x_{i - 1} } \right) \cdot y_{i - 1} + \frac{1}{2}\left( {x_{i} - x_{i - 1} } \right)\left( {y_{i} - y_{i - 1} } \right)$$

The AUC were approximated by simple integral (alike to trapezoidal integration) where the first term defines a rectangle and second term denies a tringle^24^.

### Mutational effect on allostery

Protein structure was prepared for point mutation using Chimera rotamers tool (In structure editing section)^29,49^. Upon mutation, residue conformation was chosen according to highest probability from the rotamer library (probability is taken from the library that was not affect the structural environment but changed in Phi and Psi angle when Dunbrack library was used). We have chosen two mutations (Ser441Leu and Arg217Cys) from different hotspot position (regulatory NADH and GTP binding site) with highest frequency of HI/HA mutations according to Stanley et al.^15^. Then we mutated Gly376 with possible replacement having seen in HI/HA syndrome patent (Arg, Cys, Ser, Asp, Val) and we were chosen Asp for mimicking the mutational effect on structural rigidity with Ser441Leu and Arg217Cys. After mutation, each structure minimized for removing classes and contact up to 1000 steps using Steepest decent method with step size 0.02 Å and none of the atom was fixed. After adding hydrogen, we also added charges using Amber ff99SB force field. We also defined ligand charges using ANTICHEMBER^50^ for minimization. All this process was completed using chimera Minimize Structure tool. Before used this mutated structure for further analysis, we removed all the hydrogen from the structure to maintain the method of adding polar hydrogen. To understand the effect of mutation on allosteric transmission, transmission of allosteric intensity was applied with RTA and changes in rigid clusters using FIRST (compared with wild type with mutated structures using FIRST at hydrogen bond energy strength − 1.0 kcal/mol), we carried out same process (as described above) on all mutated structures (Gly376Asp, Ser441Leu and Arg217Cys). Well-tempered metadynamics simulation^51^ was performed up to 10 ns on Gly376Asp by GROMACS patched with PLUMED v2.2.3^22^ to understand the high energy pass and protein geometry upon mutation (Details in Supplementary).

## Supplementary information

Supplementary Information.

## Data Availability

All the figure-specific codes are uploaded at github.com/soumenbera89.
