# Supplementary material for: Allosteric regulation of glutamate dehydrogenase deamination activity
Source: Sci Rep. 2020 Oct 5;10:16523. doi: 10.1038/s41598-020-73743-4 (PMC7536180; doi:10.1038/s41598-020-73743-4)
Supplement: Supplementary file 1 — Supplementary Information. [file 41598_2020_73743_MOESM1_ESM.pdf]

Supplementary Information

Allosteric regulation of Glutamate dehydrogenase deamination activity

Soumen Bera[1†], Mubasher Rashid[1], Alexander B. Medvinsky[2], Gui-Quan Sun[3,4*], Bai-Lian Li[5], Claudia Acquisti[6], Adnan Sljoka[7,8†], Amit Chakraborty[1]**

[1]School of Mathematics, Statistics and Computational Sciences, Central University of Rajasthan, Bandarsindri, Ajmer, India.

[2]Institute of Theoretical and Experimental Biophysics, Pushchino, Russia.

[3]Department of Mathematics, North University of China, Shanxi, People's Republic of China.

[4]Complex Systems Research Center, Shanxi University, Shanxi, People's Republic of China

[5]Department of Botany and Plant Sciences, University of California, Riverside, USA.

[6]Institute for Theoretical Biology, Humboldt University, Berlin, Germany.

[7]RIKEN Center for Advanced Intelligence Project, Tokyo, Japan.

[8]Department of Chemistry, University of Toronto, Toronto, Canada.

****Corresponding author:**
Amit Chakraborty
E-mail: amitc.maths@curaj.ac.in

***Co-corresponding author:**
Gui-Quan Sun
E-mail: gquansun@126.com

Lead contact:
Amit Chakraborty
E-mail: amitc.maths@curaj.ac.in

[†]both the authors contributed equally.

Table S1. Detail structural information of GDH helices

Helix No	GDH Apo (Helix size)			NADH.GTP.GDH open (Helix size)			NADH.GTP.GDH closed (Helix size)			Correlation Coefficient(r)
	Residue Id	Length (Å)	Radius (Å)	Residue Ids	Length (Å)	Radius (Å)	Residue Ids	Length (Å)	Radius (Å)	
α1	8-29	33	2	8-32	35.5	2.4	8-30	34.1	2.3	0.79577
α2	38-53	25.2	1.8	39-53	23.5	1.9	37-53	25.7	1.9	
α3	100-115	23.6	1.9	100-118	28.1	1.9	100-118	27.6	1.9	
α4	139-154	24.9	1.9	139-155	26.1	1.8	139-155	26.1	1.8	
α5	172-184	19.7	1.9	172-184	19.6	1.9	172-187	23.2	2.0	
α6 (intermediate of pivotal, α8 and α9 helix)	213-237	32.6	2.7	213-224	19.2	1.8	213-237	32.2	2.7	0.25427
α7	--------			229-234	8.1	2.0	-----------			
α8	253-265	18.7	1.9	255-265	17.3	1.8	253-265	19.1	1.9	
α9	287-299	16.9	2.1	287-298	16.4	2.0	287-298	16.1	2.0	
α10	353-361	14.2	1.8	353-363	16.1	1.8	353-363	15.9	1.9	
α11 (elongated helix)	376-391	23.5	1.9	369-388	31.5	2.0	369-388	31.2	2.0	
α12 (Antenna helix)	398-425	38.7	2.4	398-407	15.9	1.8	398-425	42.2	2.3	0.21784
α13 (Antenna helix)	---------			409-421	19.3	1.8	----------			
α14 (Antenna helix)	434-442	13.1	1.9	433-442	13.6	2.0	433-439	10	1.9	
α15 (pivotal helix)	444-471	42.1	1.9	444-471	42.2	1.9	444-471	41.1	1.9	0.79394
α16	476-497	33.3	1.9	476-497	33.2	1.9	476-496	33.1	1.9	0.03860

This table presents the helix number, helix length, radius and corresponding correlation coefficient (r) in comparison with the open form of NADH.GTP.GDH complex structure.

Helix number is computed from the Stride Services[1]. Helix length and radius are calculated from the Chimera software. Low deviation correlation coefficient indicates the lack of similar pattern between helices (comparing the open form with the closed form). Table indicates α1-α5 and α15 have high level of correlation compared to the helices α6 to α11 and Antenna helices. Moreover, there is a lack of similarity in the NBD (α6 – α11) and Antenna regions.

Notes: Deviation of Cα atoms:

Cα deviation movement is measured using the following formula:

$$\sqrt{\frac{1}{n}\sum_1^n((x_i - x_0)^2 + (y_i - y_0)^2 + (z_i - z_0)^2)}\ ,$$

where (x_i, y_i, z_i) are the position of the Cα atoms of every residue within GDH (pdb ids: 3jd3 and 3jd4). The deviation is measured in all the cases including the apo GDH structure (pdb id: 3jcz). Then the deviations are compaired and visualized with the different conformation of GDH using Python coding. Helix numbers are also included with this comparison to indicate the region of changes in Cα deviation.

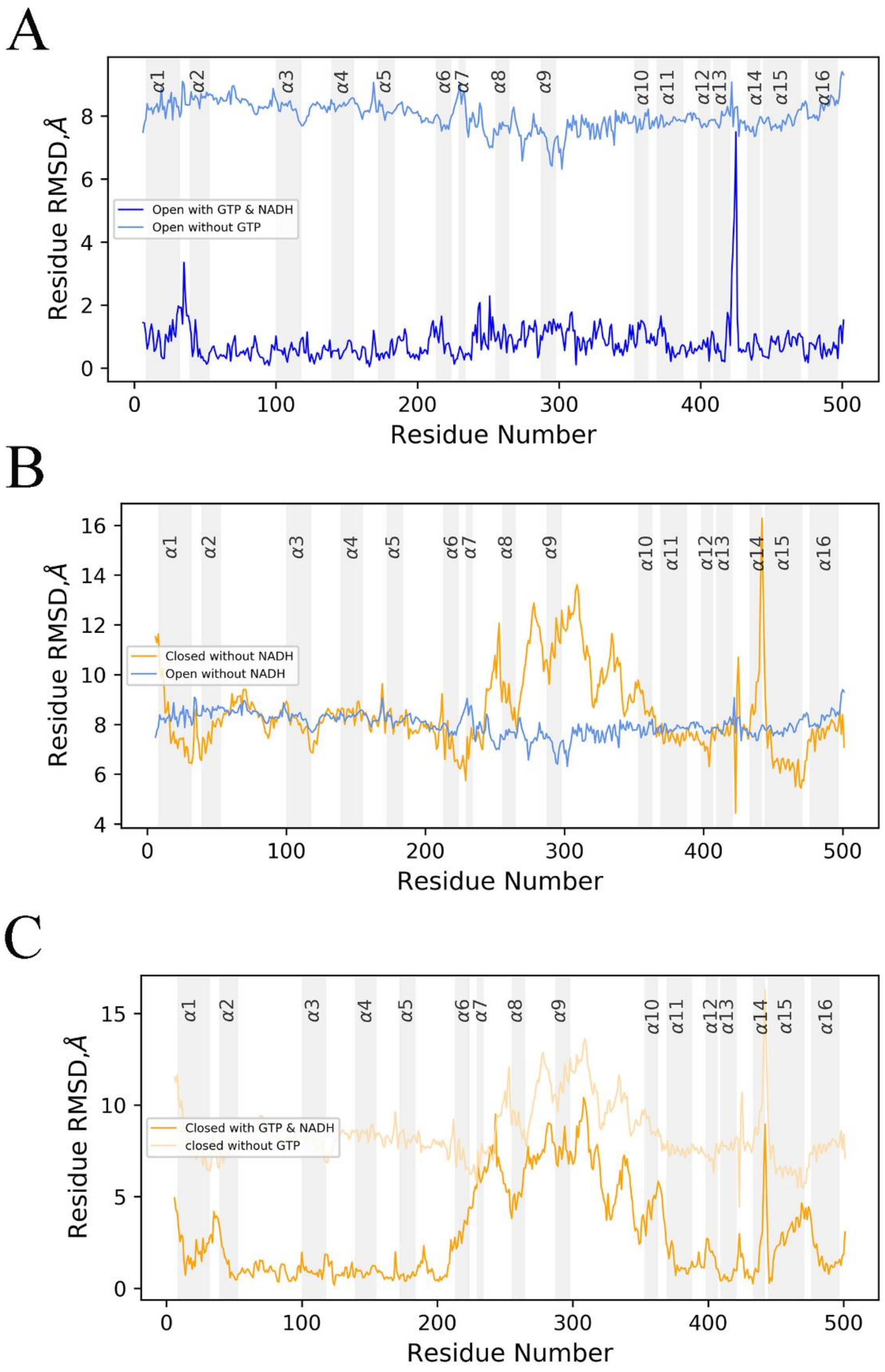

Fig. S1. Comparison of Cα-deviation.

It shows the individual Cα deviation of different structures (GDH with NADH closed form, GDH with NADH open form, GDH with NADH and GTP open and GDH with NADH and GTP closed forms) superimposing with the Apo structure (GDH only). (A) Plot indicates the effects of the GTP on open structures, (B) It also indicates the effects of GTP on the open as well as on the closed forms, (C) Plot compares individual residue deviation between closed

form structures with and without GTP. Helices are indicated with shaded region and all the figures are generated using Python coding.

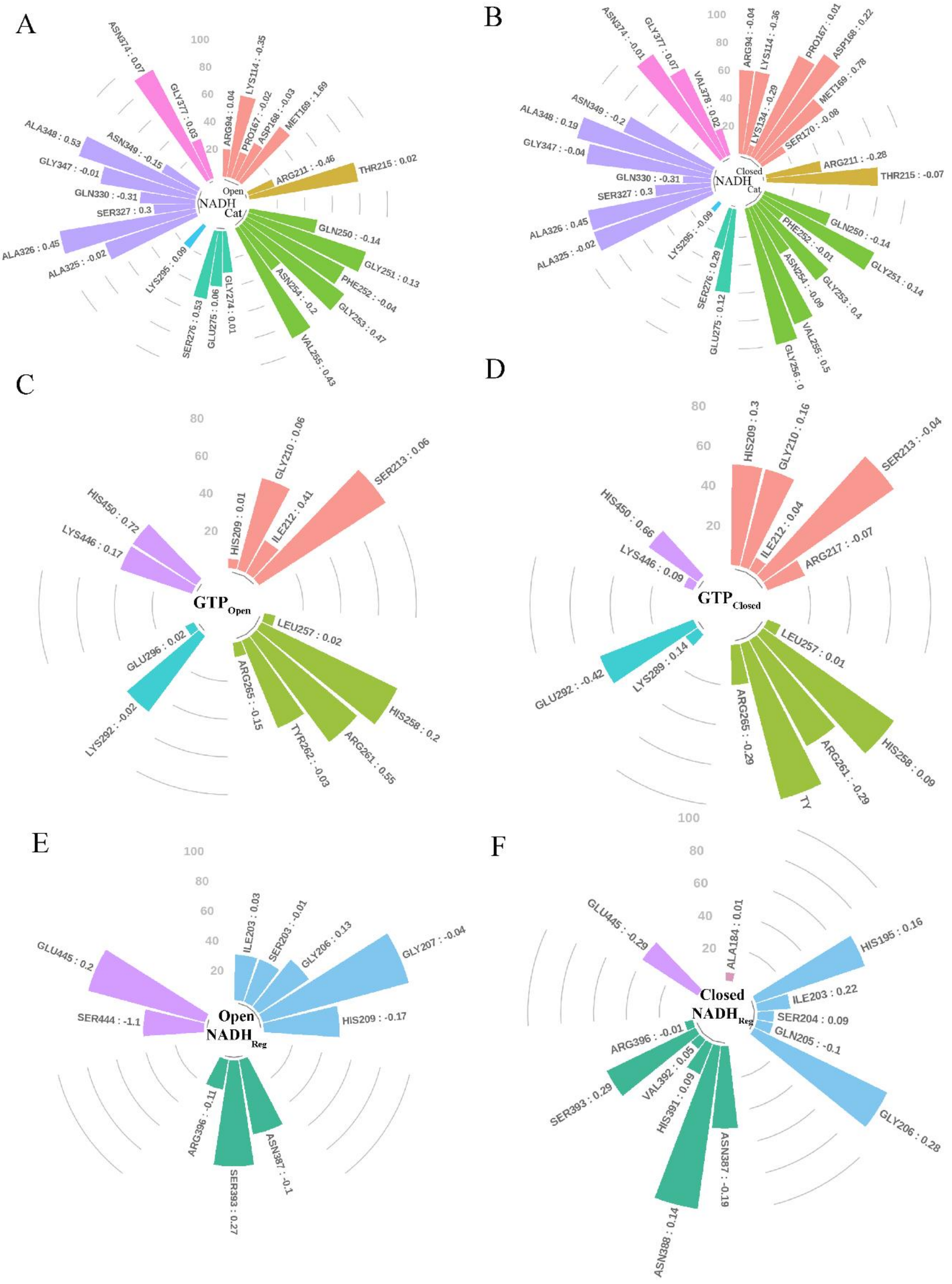

Fig. S2. Interaction between the cofactors NADH, inhibitor GTP with the GDH.

Plot showing details information of interface residues with the ligands at two different conformations (open and closed). Group wise colours designates with alpha helices or outside

of alpha helices. Each bar represents the contribution of buried surface area of interfacing residues with ligands, and the fraction numbers at top of the head of each bar is showing solvation energy effect ($\Delta^{i}G$ Kcal/mol). (A), (B) figures are showing the interaction between interface residues of protein and catalytic NADH at open and closed confirmation. Number of interfacing residues (29) in the closed conformation is much higher than the open (26) form at the catalytic site. Medium purple colour bars indicate the residue in the NBD domain, but not in the alpha helices. Whereas violate and lime green bars show residues situated at α11 and α8 respectively. (C), (D) represents the interfacing residues of protein with the GTP. Percentage of buried surface area of His 209, Arg261 and Tyr 262 has sufficiently increased in closed structure compare to the open form. (E) and (F) represent the interfacing residues of GDH with NADH in the open and closed forms, respectively.

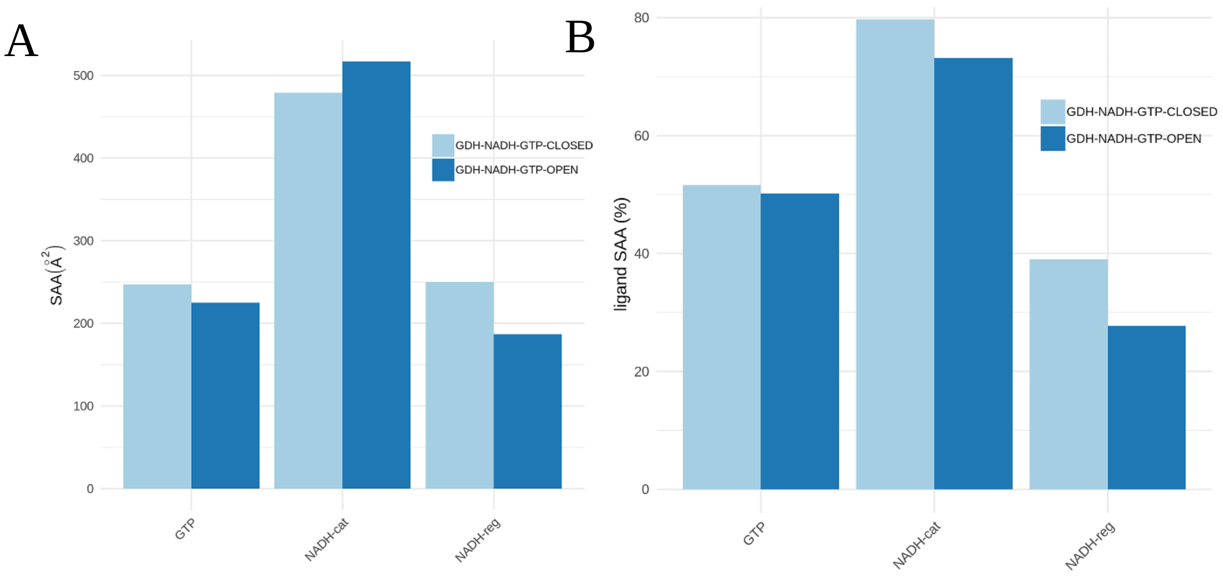

Fig. S3. Solvent Accessible Area of protein and ligand in-between the protein ligand interfaces.

(A) It represents Solvent Accessible Area^2 of GDH protein interfaces with GTP, NADH catalytic and NADH regulatory sites, (B) is showing the SAA (%) of GTP, NADH catalytic and NADH regulatory interfaces. The solvent accessibility of protein at NADH catalytic site is significantly higher for the open structures, which indicates the flexibility of protein at catalytic site in compared to the closed one.

Table S2. Comparison between protein and ligand interfaces

Structure1				Structure2						
**Range**	N_{at}	N_{res}	Surface (Å^2)	Range	Nat	Nres	Surface (Å^2)	Interface Area(Å^2)	ΔG Kcal/mol	N_{HB}
**Chain A**	73	28	23318	NAI: Catalytic	42	1	825	560.0	-7.8	8
**Chain C**	30	10	23318	NAI: Regulatory	20	1	773	200.8	-1.9	2
**Chain A**	34	13	23318	GTP	24	1	540	248.0	-5.4	3

3jd3 open structure: Interface

Structure1				Structure2						
**Range**	N_{at}	N_{res}	Surface (Å^2)	Range	Nat	Nres	Surface (Å^2)	Interface Area(Å^2)	ΔG Kcal/mol	N_{HB}
**Chain A**	80	31	22631	NAI: Catalytic	43	1	802	559.2	-6.4	8
**Chain A**	40	14	22631	NAI: Regulatory	28	1	829	286.9	-3.4	2
**Chain A**	34	14	22631	GTP	26	1	573	271.4	-1.4	7

3jd4 closed structure: Interface

The above table summarizes the interfaces of **open form structure (pdb id: 3jd3) and closed form structure (pdb id: 3jd4)**. N_{at}, N_{res} indicate the number of atoms and the number of residues respectively involved in the interface area. N_{HB} indicates number of hydrogen bonds present at the interfaces and ΔG (Kcal/mol) is the solvation energy for folding. All information was extracted from PDBePISA (https://www.ebi.ac.uk/pdbe/pisa/).

Table S3. Identifying hydrogen bond at the interfaces

A **Catalytic NADH interface: Hydrogen bonds**

No	Structure1	Dist (Å)	Structure2
1	A: ASN 254 [N]	2.92	A: NAI 602 [O2A]
2	A: PHE 252 [N]	2.88	A: NAI 602 [O3B]
3	A: GLY 253 [N]	3.11	A: NAI 602 [O3B]
4	A: SER 276 [N]	2.47	A: NAI 602 [O2B]
5	A: ASN 254 [N]	2.76	A: NAI 602 [O3]
6	A: ASN 254 [ND2]	3.03	A: NAI 602 [O2N]
7	A: THR 215 [OG1]	3.89	A: NAI 602 [O7N]
8	A: GLN 250 [O]	3.61	A: NAI 602 [N3A]

Regulatory NADH interface: Hydrogen bonds

No	Structure1	Dist (Å)	Structure2
1	A: SER 444[OG]	3.40	C: NAI 601[O3D]
2	A: HIS 209[NE2]	3.84	C: NAI 601[O2D]

B **Catalytic NADH interface: Hydrogen bonds**

No	Structure1	Dist (Å)	Structure2
1	A: SER 170 [N]	2.92	A: NAI 601 [O1A]
2	A: ASN 254 [N]	3.26	A: NAI 601 [O2A]
3	A: ASN 254 [N]	3.68	A: NAI 601 [O3]
4	A: ASN 254 [ND2]	3.54	A: NAI 601 [O2N]
5	A: ARG 94 [NH2]	3.85	A: NAI 601 [O3D]
6	A: ASN 349 [N]	3.78	A: NAI 601 [O2D]
7	A: ARG 94 [NH2]	3.03	A: NAI 601 [O2D]
8	A: ASN 349 [ND2]	2.45	A: NAI 601 [O2D]

Regulatory NADH interface: Hydrogen bonds

No	Structure1	Dist (Å)	Structure2
1	A: VAL 392[N]	3.36	C: NAI 603[O3D]
2	A: HIS 195[NE2]	3.32	C: NAI 603[O2D]

(A) It represents the GDH.GTP.NADH closed form structure and (B) represents the GDH.GTP.NADH open form structure. The numbers of hydrogen bonds at the GDH and NADH interfaces are same in both the forms; however, there are significant changes in amino acids and corresponding hydrogen bond distances (Å). Moreover, in the closed form, catalytic NADH is showing stronger interaction with the NBD domain (all the hydrogen bond formation amino acid belongs to the NBD domain).

Table S4. Identifying hydrogen bond in the GTP-GDH interfaces.

A **GTP binding: Hydrogen bonds**

No	Structure1	Dist (Å)	Structure2
1	A: ARG 265 [NH1]	3.60	A: GTP 602 [O1G]
2	A: ARG 265 [NH1]	3.53	A: GTP 602 [O2G]
3	A: TYR 262 [OH]	2.35	A: GTP 602 [O3G]
4	A: HIS 209 [NE2]	2.36	A: GTP 602 [O2B]
5	A: SER 213 [OG]	2.75	A: GTP 602 [O2]
6	A: GLU 292 [OE1]	3.43	A: GTP 602 [N1]
7	A: GLU 292 [OE2]	2.72	A: GTP 602 [N2]

B **GTP binding: Hydrogen bonds**

No	Structure1	Dist (Å)	Structure2
1	A: ARG 261 [NH2]	2.78	A: GTP 601 [03G]
2	A: TYR 262 [OH]	2.84	A: GTP 601 [O3G]
3	A: HIS 450 [NE2]	3.23	A: GTP 601 [O1B]

C **GTP without NADH: Hydrogen bonds**

No	Structure1	Dist (Å)	Structure2
1	A: SER 213 [OG]	3.88	A: GTP 601 [O1G]
2	A: ARG 261 [NE]	2.74	A: GTP 601 [O3G]
3	A: ARG 265 [NH2]	3.20	A: GTP 601 [O3G]
4	A: ARG 261 [NH2]	2.71	A: GTP 601 [O3G]
5	A: GLU 292 [OE2]	3.13	A: GTP 601 [N1]

(A) and (B) represent hydrogen bonds with the GTP in GDH closed and open form structures, respectively. (C) represents the number of hydrogen bonds with the GTP in GDH.GTP complex without NADH. In the closed structure, it shows a high number of hydrogen bonds compared to the GDH.GTP open form structure.

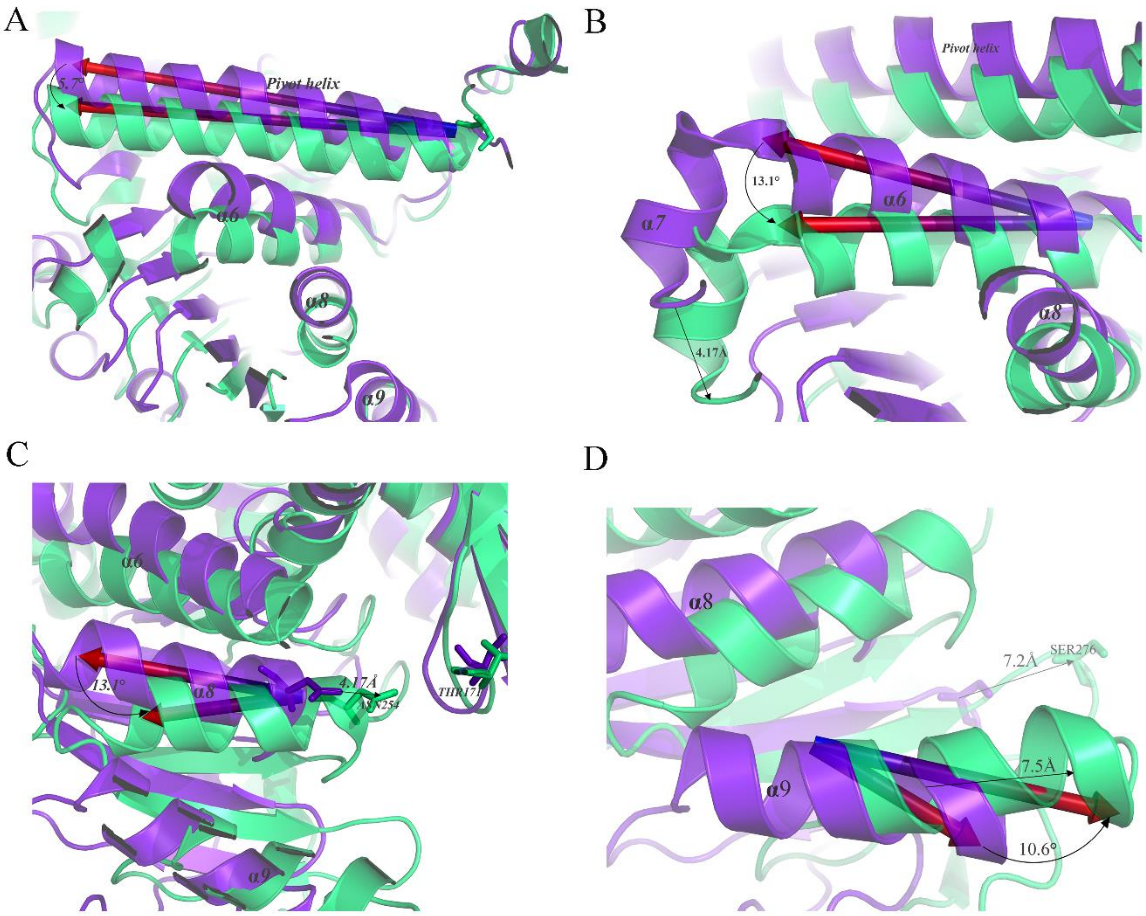

Fig. S4. **Translation and rotation of alpha helices**

This superimposed (open form structure is represented in purple and closed form in lime green colour) helices (residue ids from 200 to 390) within the GDH structures show significantly higher RMSD compared to the others. (A) and (B) show anti clockwise rotation of pivotal and α6 helices of 5.7^{o} and 13.1^{o} respectively. (C) and (D) represent α8 and α9 rotation of 13.1^{o} and 10.6^{o}, respectively, while they are translated 4.75Å and 7.5Å towards the catalytic site. This translation as well as rotation indicates the shifting of Ser276 (approx. 7.2Å) of NBD domain towards the catalytic site and then it closed the catalytic cleft. All the figures are generated in UCSF Chimera[3].

Table S5. Mutational hotspots region.

Number of mutations	Amino acid change	"Sporadic"/ "Familial"	Exons	Positions in The structure
1	SER → Cys at 213	0/1	6 and 7	4 Å of GTP
12	Arg → Cys at 217	3/9	6 and 7	4 Å of GTP
2	His → Thr at 258	2/0	6 and 7	4 Å of GTP
2	Arg → Thr at 261	2/0	6 and 7	4 Å of GTP
1	Arg → Ser at 261	0/1	6 and 7	4 Å of GTP
1	Tyr → Cys at 262	1/0	6 and 7	4 Å of GTP
1	Tyr → His at 262	1/0	6 and 7	4 Å of GTP
13	Arg → His at 265	2/11	6 and 7	4 Å of GTP
1	Arg → Cys at 265	1/0	6 and 7	4 Å of GTP
1	Leu → Val at 409	1/0	10,11 and 12	Antenna
2	Asn → Tyr at 406	2/0	10,11 and 12	Antenna
1	Phe → Leu at 436	1/0	10,11 and 12	Antenna
1	Gln → Arg at 437	1/0	10,11 and 12	Antenna
4	Gly → Val at 442	4/0	10,11 and 12	Antenna
3	Gly → Asp at 442	3/0	10,11 and 12	Antenna
3	Gly → Ser at 442	3/0	10,11 and 12	Antenna
1	Gly → Cys at 442	1/0	10,11 and 12	Antenna
1	Gly → Arg at 442	1/0	10,11 and 12	Antenna
25	Ser → Leu at 441	24/1	10,11 and 12	Antenna
1	Ala → Thr at 443	0/1	10,11 and 12	Antenna
3	Ser → pro at 444	0/3	10,11 and 12	Pivotal
2	Lys → Glu at 446	1/1	10,11 and 12	Pivotal
2	His → Tyr at 450	1/1	10,11 and 12	Pivotal

This table shows the location and frequency of hyperinsulinism–hyperammonemia syndrome (HI/HA) associated mutations in the GDH. Out of the total 84 cases, 66% is sporadic and 34% familial[4,5]. The table also indicates the three mutational hotspot regions within the protein: GTP binding sites, Antenna, and the Pivotal helices region; however, the highest number of mutations in HI/HA cases has been observed at Leu441 whose position is in the junction of pivotal and Antenna helices. Another position of mutation is His265 whose frequency is also significantly higher in the HI/HA cases.

Table S6. Rigid cluster and flexibility analysis using ProFlex/FIRST

Structure ID	Hydrogen bond energy strength	Highest size rigid cluster	Participating helices (%, towards N or C terminal) within rigid cluster	Total No of Rigid Cluster	Independent DOF	No of hydrogen bond	Independent Hinge joint
3jczA (Open)	0.0	5274	$\alpha1(91,N), \alpha2(93,C), \alpha3, \alpha4, \alpha5, \alpha6\&\alpha7(0.87,C)$ $\alpha8(67,C), \alpha9(92,N), \alpha10, \alpha11(93,N), \alpha15, \alpha16(90,N)$	1264	614	393	476
	0.5	4791	$\alpha1(86,N), \alpha2(33,C), \alpha3, \alpha4, \alpha5, \alpha6\&\alpha7(87,C)$ $\alpha8(67,C), \alpha10, \alpha11(93,N), \alpha15, \alpha16(90,N)$	1448	663	349	506
	1	1867	$\alpha4(20,N), \alpha5, \alpha6\&\alpha7(83,C), \alpha8(67,C), \alpha10, \alpha15,$ $\alpha16(67,N)$	2265	740	297	544
	1.5	1362	$\alpha6\&\alpha7(45,N\&C), \alpha8(67,C), \alpha10, \alpha16(52,N)$	2721	836	251	567
	2	805	$\alpha6\&\alpha7(45,N\&C), \alpha8(67,C)$	3009	909	220	585
3jd3A (Open)	0.0	4809	$\alpha1, \alpha2, \alpha3, \alpha4, \alpha6\&\alpha7(95,C), \alpha8, \alpha9(73,N), \alpha10(70,N)$ $\alpha11, \alpha15, \alpha16(90,N)$	1389	669	376	506
	0.5	2846	$\alpha1(96,N), \alpha6\&\alpha7(95,C), \alpha8, \alpha9(73,N), \alpha10(70,N)$ $\alpha11(33,N), \alpha15, \alpha16(90,N)$	1935	725	324	536
	1	1471	$\alpha6\&\alpha7(83,C), \alpha8, \alpha9(64,N), \alpha15(92,C)$	2479	809	280	570
	1.5	1025	$\alpha6\&\alpha7(62,C), \alpha8, \alpha9(64,N)$	2690	895	236	588
	2.0	232	$\alpha6\&\alpha7(38,C)$	3207	1017	190	618
3jd4A (Closed)	0.0	5580	$\alpha1(96,N), \alpha2, \alpha3(94,C), \alpha4, \alpha5, \alpha6\&\alpha7, \alpha8, \alpha9$ $\alpha10, \alpha11(95,N), \alpha15(67,C), \alpha16(92,N)$	1167	617	412	484
	0.5	5121	$\alpha1(96,N), \alpha2, \alpha3(94,C), \alpha4, \alpha5, \alpha6\&\alpha7(96,C), \alpha8$ $\alpha9(75,N), \alpha10, \alpha11(84,N), \alpha15(66,C), \alpha16(92,N)$	1355	670	363	511
	1	4784	$\alpha1(96,N), \alpha2, \alpha3(94,C), \alpha4, \alpha5, \alpha6\&\alpha7(96,C), \alpha8$ $\alpha9(75,N), \alpha10, \alpha11(84,N), \alpha15(66,C), \alpha16(92,N)$	1499	719	317	537
	1.5	1748	$\alpha3(94,C), \alpha4, \alpha5(92,N), \alpha11(43,N)$	2478	803	270	551
	2	652	$\alpha4(87,N)$	2908	903	226	580

Hydrogen bond energy strength is considered from 0 to -2.0, as the communication is started after hydrogen bond cut off 0.0 and has been stopped before the cut-off of -1.5 (Figure S5). The table also indicates the position and percentage of helices that are involved within the rigid cluster at the different hydrogen bond energy cut-off.

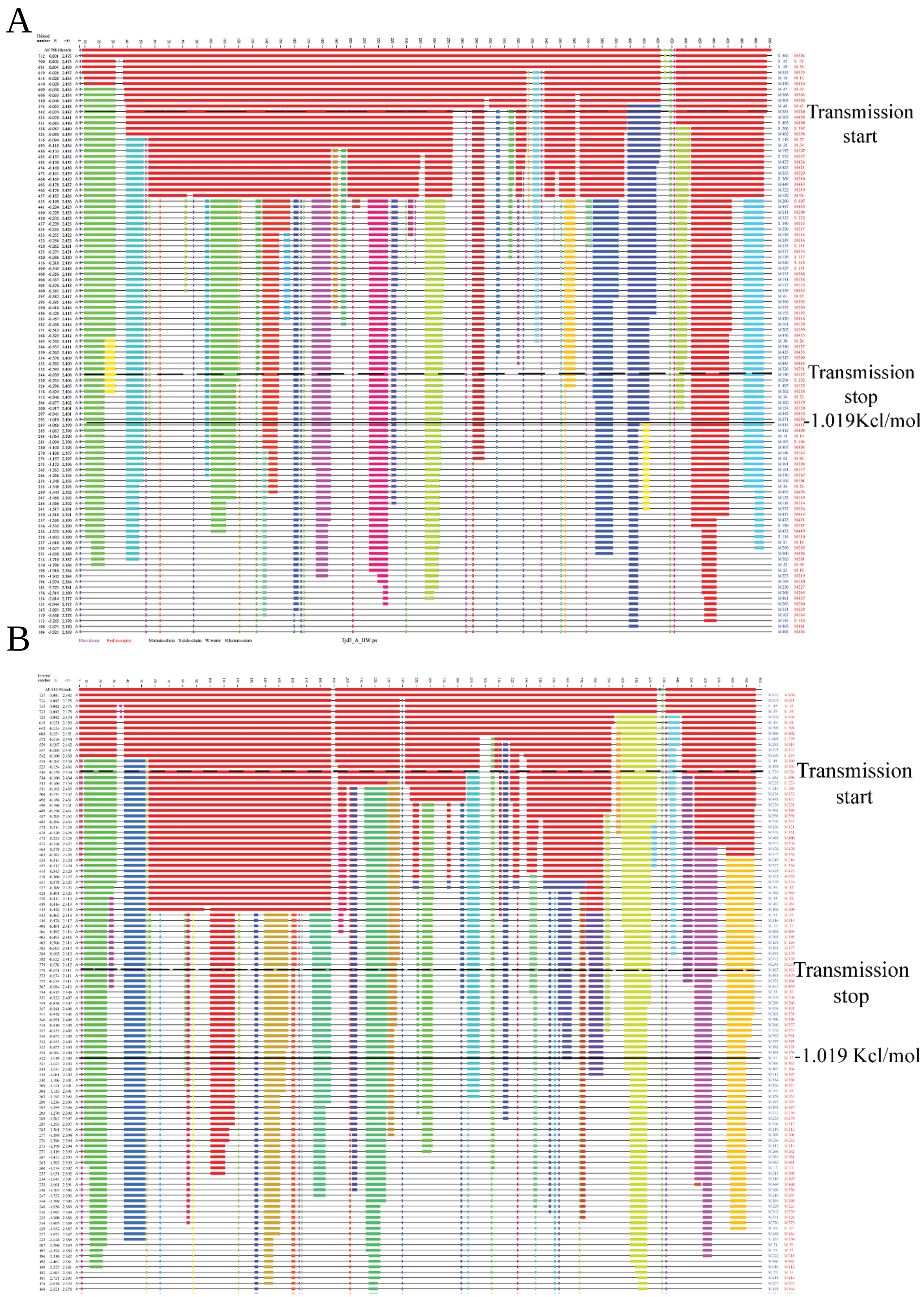

Fig. S5. Hydrogen bond dilution plot.

(A), (B) H-Dilution plot of GDH open from (PDB ID 3jd3) and GDH closed form (PDB ID 3jd4) generated in ProFlex / FIRST[6]. Horizontal axis represents residue numbers and vertical

axis represent hydrogen bond energy cut off (Kcal/mol). Protein flexibility shows by horizontal grey lines; however, the solid colour lines indicate rigid cluster at different hydrogen bond energy cut offs. Last two columns (blue and red) represent hydrogen donor and acceptor, respectively. Black dotted lines (upper and lower) indicate allosteric communication start and stop associated with the hydrogen bond energy cut off.

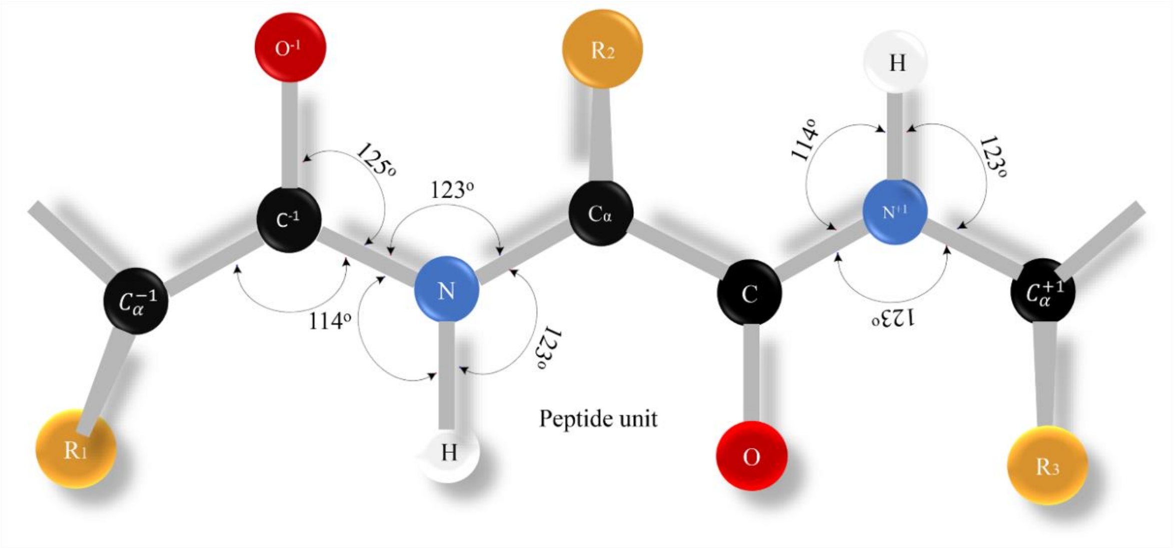

Fig. S6. Standard tripeptide chain geometry

Amino acids in proteins (or polypeptides) are joined together by peptide bonds. R_1, R_2 and R_3 group represent the side chain of each amino acid.

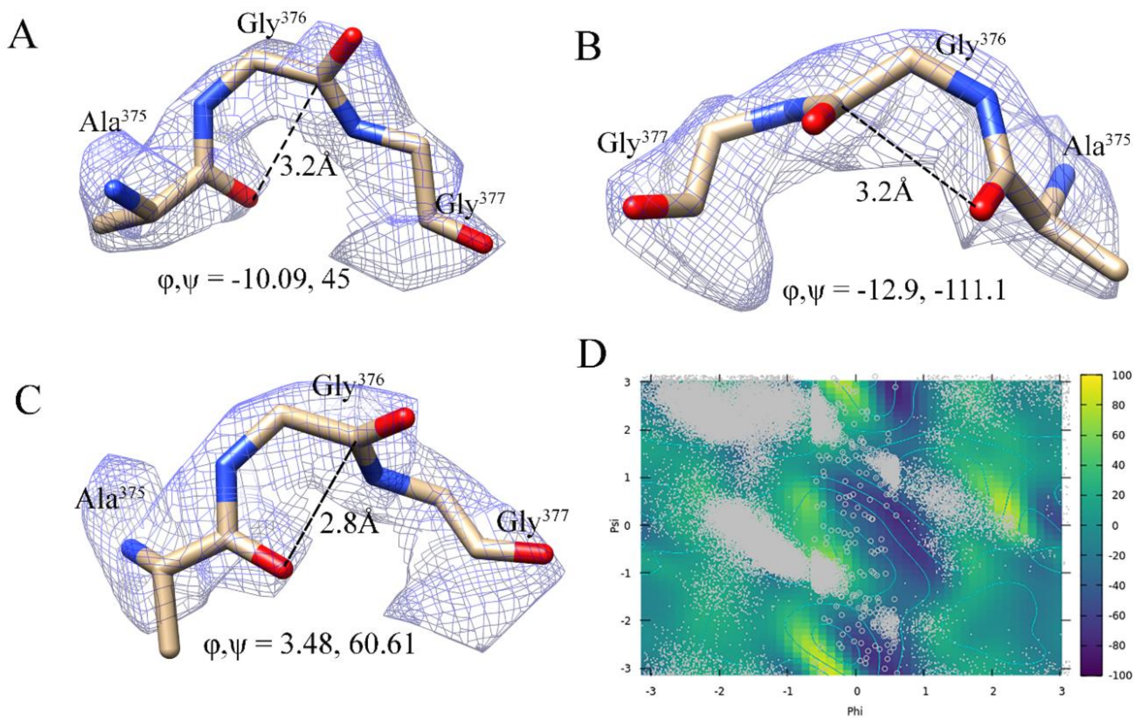

Fig. S7. **Transient Residue reliability and populated high-energy passes upon mutation.**

(A), (B) and (C) are showing the evidence of density map for a reliable transient residues at different time point of the trajectory (0.4ns, 9.3ns and 4ns). The transient resides are located at the unallowable regions of the Ramachandran plot i.e., $-35^0 < \varphi < 35^0$. The residues Ala375, Gly376 and Gly377 from the MD snapshots at different time points (0.4ns, 9.3ns and 4ns) fitted with the EM maps by chimera tools. Dotted black lines within the figure is showing O^{-1} …C distance corresponding to φ, ψ angles at different conformational states. (D) φ, ψ angles describing the local conformation changes of mountain pass residues at different time points of the MD trajectory after 10ns MD simulation with the mutation (Gly376Asp). The zoomed circle indicates the position of transient residues at $-35^0 < \varphi < 35^0$. Colour gradient also specifies the region of free energy landscape of Gly376 upon mutation by Asp.

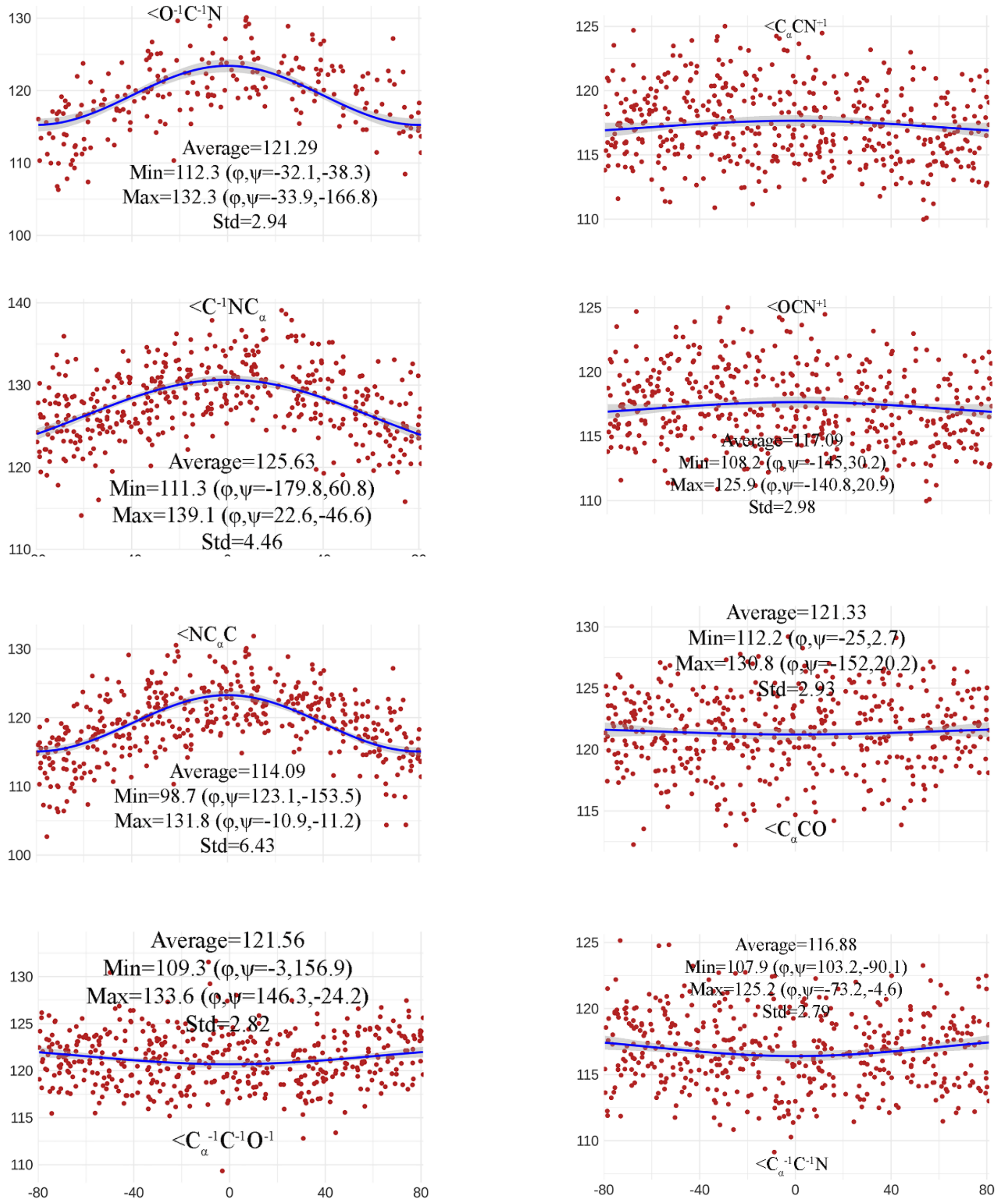

Fig. S8. Deformation of the tripeptide geometry during the transition through the high-energy passes.

Bond angle variations are calculated for the transition from the $\varphi \leq 0^o$ to $\varphi \geq 0^o$ with the MD trajectory data. Blue lines represent calculated fits of observed MD trajectory data using cosine equations. This fitted smooth curve describes the systematic distortion of bond angles.

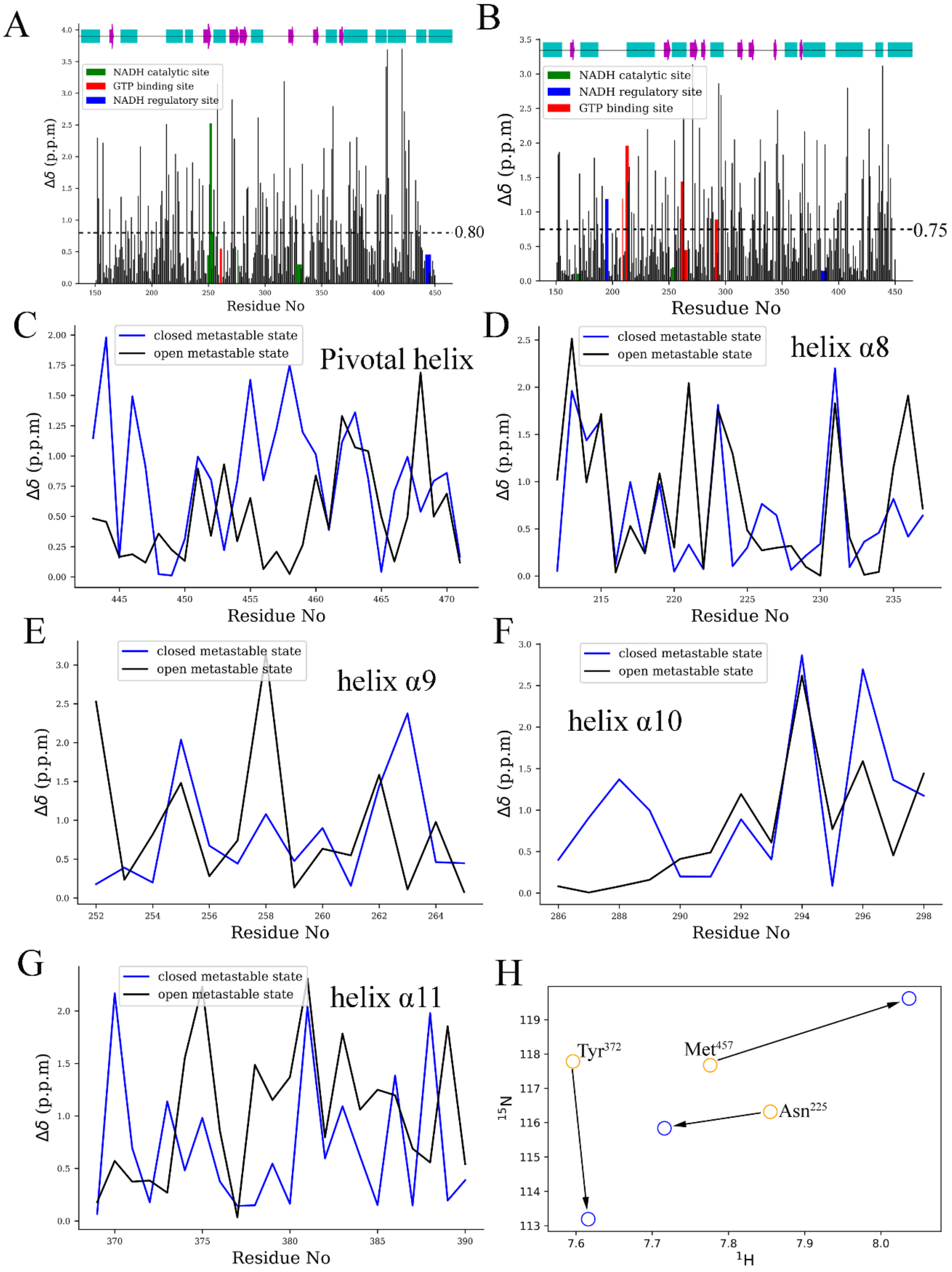

Fig. S9. Comparison of the chemical shift.

Comparison of Chemical Shifts between open and closed conformations are predicted by SHIFTX2 software[7]. (A) figure shows the difference of chemical shift between the Apo (pdb id: 3jcz) and the GDH.NADH.GTP open (pdb id: 3jd3) structure. Colour area indicated the binding site of GTP and NADH. Rectangle and arrows filled with cyan and magenta colours mapped with alpha helices and beta sheets respectively to indicate the specific position of the

residue. The dotted black line shows the average value of the difference of chemical shift (in ppm). (B) This figure shows the difference chemical shift between Apo and GDH.NADH.GTP closed (3jd4) structures. Here the average chemical shift difference (in ppm) is slightly lower than the former one. (C) shows 13CA chemical shift difference of pivotal helix where black and blue line represents NADH.GTP open and NADH-GTP closed form structures. (D), (E), (F) and (G) also show the 13CA chemical shift differences of α8, α9, α10 and α11 respectively. (H) 15N – 1H chemical shift differences of Tyr 372, Met 457 and Asn 225 that indicate the loss of hydrogen bond in open structure (figure 2). All data were generated by SHIFTX2 (http://www.shiftx2.ca/) using experimental pH and temperature(K) available in PDB data[8].

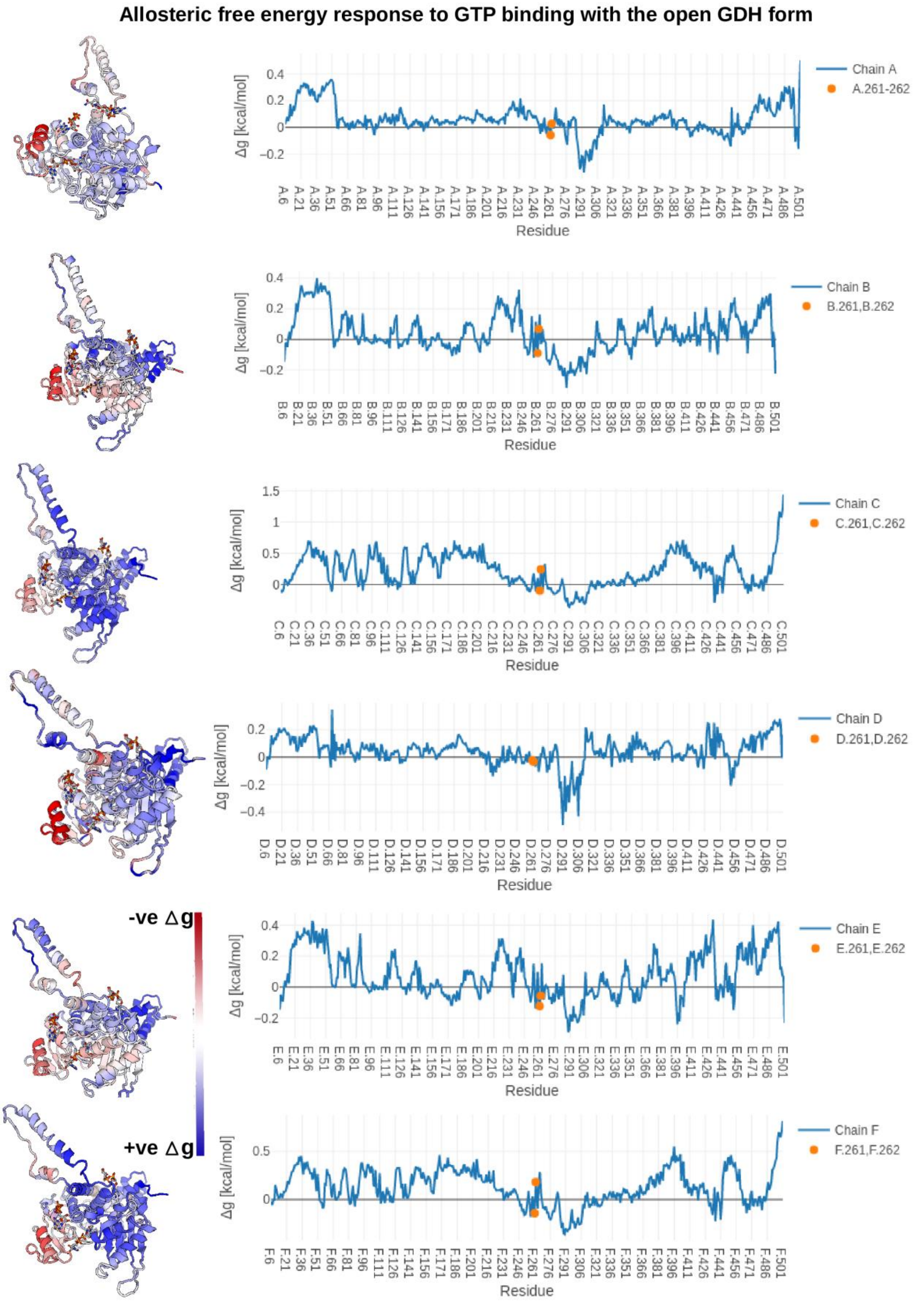

Fig.S10. *AlloSigMA* webserver is used for predicting allosteric communications between allosteric sites GTP, $NADH_{reg}$, and the catalytic sites triggered by GTP-binding for the GDH open conformer. It estimates per-residue allosteric free energies resulting from GTP and

NADH$_{reg}$ binding. Positive sign shows the effects of local destabililization, whereas negative sign indicates effects of local stabilization.

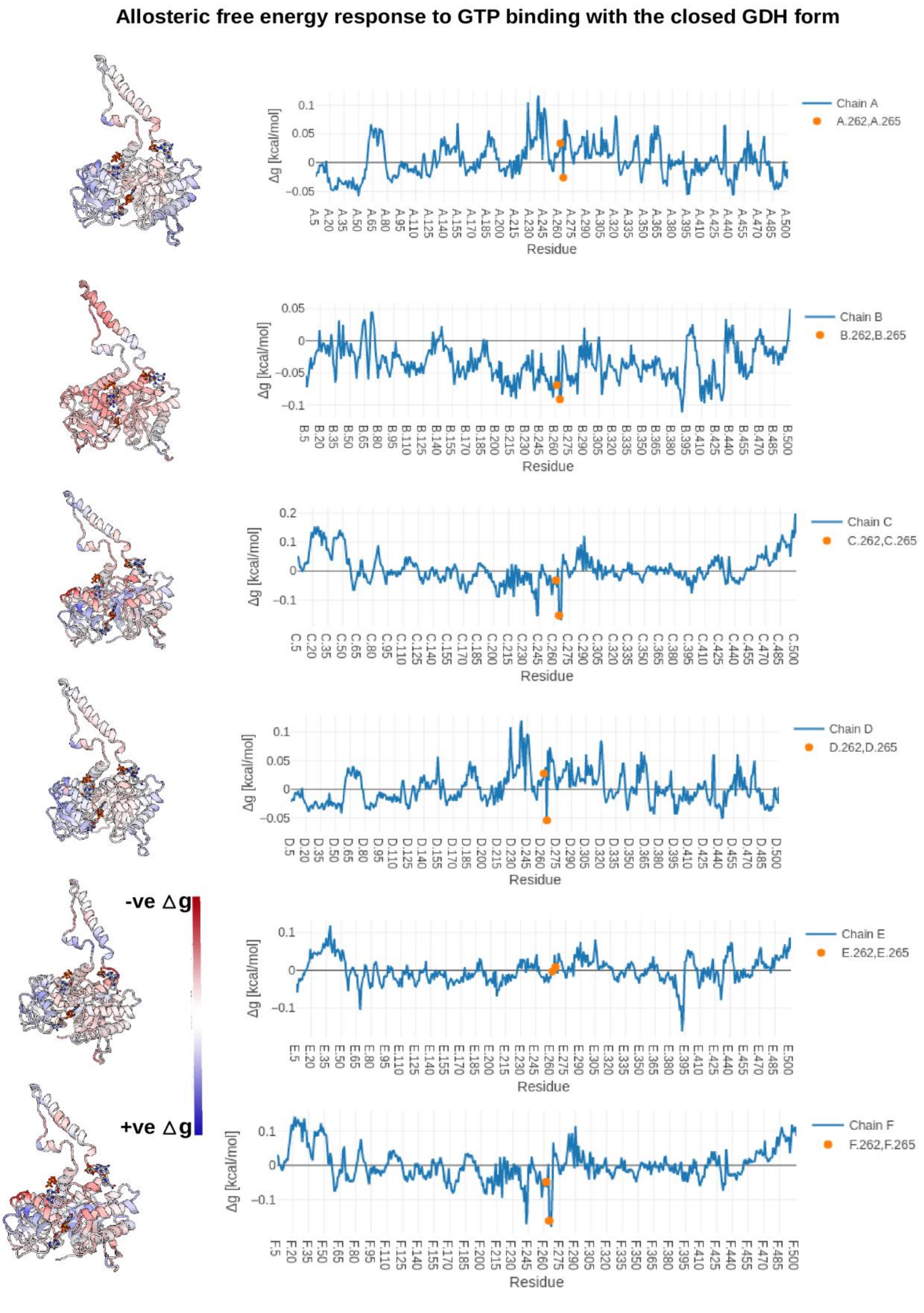

Fig.S11. *AlloSigMA* webserver is used for predicting allosteric communications between allosteric sites GTP, $NADH_{reg}$, and the catalytic sites triggered by GTP-binding for the GDH closed conformer. It estimates per-residue allosteric free energies resulting from GTP and $NADH_{reg}$ binding. Positive sign shows the effects of local destabililization, whereas negative sign indicates effects of local stabilization.

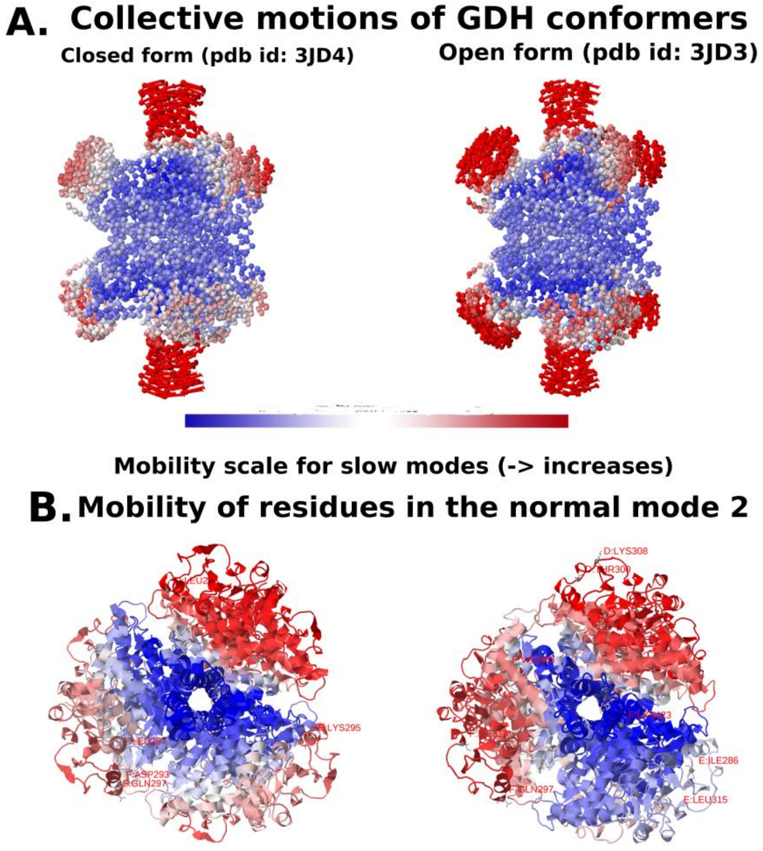

Fig.S12. Normal mode analysis (NMA) of GDH homohexamer: (A) ANM-driven collective motion showing higher structural flexibility of GDH-open conformer compared to its closed form, (B) GNM-driven collective motion representing mobility of residues across 6 monomers that shows differential domain separation within each monomer.

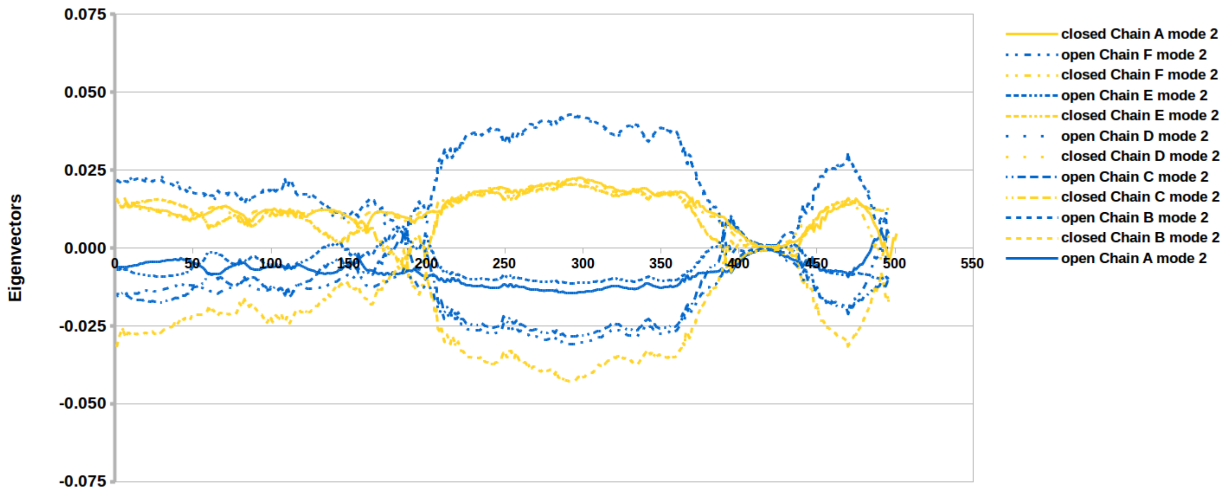

Fig.S13.Differential contributions of residues along the second global mode: these are evaluated by separating the domains based on the direction (+/-) of their movement along GNM-driven second normal mode. Residues with same sign move together in the same direction and it is predicted to form a dynamically coupled regions. It also predicts the global hinge sites located between the sequences segments that undergo opposite direction movements along the slowest mode. Across all the six monomers, it shows domain separation of NBD and regulatory sites by the allosteric regions lie within residues ~200-400.

Table S7. Frequency of amino acids in the high-energy transition region.

Reside ID &NO	Frequency >=10	Position
ALA-194	12	Regulatory
ALA-341	31	coenzyme binding domain
ALA-375	22	Catalytic&376
ALA-443	91	Regulatory
ASN-254	20	Catalytic
ASP-119	17	--------
GLU-25	42	--------
GLU-328	49	Catalytic
GLU-36	19	--------
GLY-243	68	coenzyme binding domain
GLY-350	53	Catalytic
GLY-376	197	Catalytic
GLY-377	19	Catalytic
HIS-189	24	Regulatory
HIS-209	31	Regulatory
LEU-371	14	coenzyme binding domain and alpha11
LYS-329	28	Catalytic
MET-169	63	Catalytic
PHE-122	84	---------
PHE-304	10	coenzyme binding domain
PRO-165	39	Catalytic

PRO-167	40	Catalytic
PRO-202	21	Regulatory
PRO-240	32	coenzyme binding domain
PRO-288	16	coenzyme binding domain
PRO-354	19	coenzyme binding domain
PRO-432	82	Antenna
PRO-7	56	N-terminal
PRO-88	15	-------
SER-327	33	--------
SER-393	41	Regulatory
THR-34	18	--------
THR-37	36	--------
THR-427	14	Antenna
VAL-99	19	Catalytic

Table listed all the amino acid residues with their frequency higher than 10 that fall into the region $-35^0 < \varphi < 35^0$ along the MD trajectory. Cyan colour indicates the frequency of Gly376 and its neighbouring residues.

Table S8. Frequency of amino acids in the high-energy transition region upon mutation.

Reside ID &NO	Frequency >=10	Position
ALA-336	10	coenzyme binding domain
ALA-341	43	coenzyme binding domain
ALA-434	25	antenna
ARG-211	36	GTP binding area
ARG-396	15	regulatory
ASP-119	47	------
ASP-138	12	------
ASP-370	18	coenzyme binding domain and alpha11
ASP-376	168	catalytic
GLU-173	25	catalytic
GLU-25	60	------
GLU-36	33	------
GLU-38	13	------
GLY-243	47	coenzyme binding domain
GLY-350	147	catalytic
GLY-377	16	catalytic
GLY-422	34	antenna
GLY-442	20	regulatory
ILE-203	11	regulatory
LEU-371	251	coenzyme binding domain and alpha11
LYS-329	26	------
LYS-53	20	------
MET-169	162	catalytic
PHE-304	20	coenzyme binding domain
PHE-9	18	N-terminal

PRO-165	116	catalytic
PRO-202	56	regulatory
PRO-240	30	coenzyme binding domain
PRO-354	17	catalytic
PRO-429	43	antenna
PRO-432	45	antenna
PRO-7	40	N-terminal
SER-276	58	coenzyme binding domain
SER-279	20	coenzyme binding domain
SER-393	41	regulatory
SER-83	19	None
VAL-378	24	catalytic

With Gly376Asp mutation, table listed the amino acid residues with the frequency more than 10 that lie in the high energy passes demarcated by $-35^0 < \varphi < 35^0$ along the computed MD trajectory. Gly376 and its neighbourhood residues were highlighted in cyan colour showing less frequency compared to the previous table.

Table S9. Distance measurement among the distant potential allosteric sites for the <u>open form GDH</u>

Nearest atoms distances between GTP, NADH (Regulatory), and NADH (Catalytic) sites:

Nearest atoms of GTP from NADH	Nearest atoms of NADH from GTP	Distance (Å)
GTP (O1A)	NADH-Regulatory (O2D)	7.7
GTP (N7)	NADH-Catalytic (O2A)	11.1

Distance between residues forming hydrogen bonds with GTP, NADH-regulatory, and NADH-Catalytic sites:

Closest residues formed H-bond with the GTP	Nearest residues formed H-bond with the NADH from GTP	C-alpha distance (Å)
TYR262	SER444 (NADH-Regulatory)	19.5
ARG261	ASN254 (NADH-Catalytic)	11.4

Distance between mutations and catalytic site (nearest h-bonds):

Mutations	Nearest residues formed H-bonds with the residues of the catalytic site after mutations	C-alpha distance (Å)
GLY376ASP	ASN254	12.7
ARG217CYS	ASN254	10.6
SER441LEU	ASN254	29.1

Table S10. Distance measurement among the distant potential allosteric sites for the <u>closed form GDH</u>

Nearest atoms distance between GTP, NADH (Regulatory), and NADH (Catalytic) sites:

Nearest atoms of GTP from NADH	Nearest atoms of NADH from GTP	Distance (Å)
GTP (C4')	NADH-Regulatory (O1A)	12.80
GTP (N2)	NADH-Catalytic (O2A)	11.569

Distance between residues forming hydrogen bonds with GTP, NADH-regulatory, and NADH-Catalytic sites:

Closest residues formed H-bond with the GTP	Nearest residues formed H-bond with the NADH from GTP	C-alpha distance (Å)
HIS209	ASN387 (NADH-Regulatory)	11.10
SER213	ASN254 (NADH-Catalytic)	7.6
GLU292	ASN254 (NADH-Catalytic)	10.7

* Other two hydrogen bonds TYR262, ARG265 with GTP have higher distances (in Angstrom) from the NADH binding site.

Distance between mutations and catalytic site (nearest H-bonds):

Mutations	Nearest residues formed H-bonds with the residues of the catalytic site after mutations	C-alpha distance (Å)
GLY376ASP	ASN254	13.1
ARG217CYS	ASN254	11.4
SER441LEU	ASN254	32.4

References

1 Heinig, M. & Frishman, D. STRIDE: a web server for secondary structure assignment from known atomic coordinates of proteins. *Nucleic acids research* **32**, W500-W502 (2004).

2 Marsh, J. A. Buried and accessible surface area control intrinsic protein flexibility. *Journal of molecular biology* **425**, 3250-3263 (2013).

3 Pettersen, E. F. *et al.* UCSF Chimera—a visualization system for exploratory research and analysis. *J. Comput. Chem.* **25**, 1605-1612 (2004).

4 Stanley, C. A. Two genetic forms of hyperinsulinemic hypoglycemia caused by dysregulation of glutamate dehydrogenase. *Neurochem. Int.* **59**, 465-472 (2011).

5 Stanley, C. A. *et al.* Hyperinsulinism and hyperammonemia in infants with regulatory mutations of the glutamate dehydrogenase gene. *New Engl. J. Med.* **338**, 1352-1357 (1998).

6 Jacobs, D. J., Rader, A. J., Kuhn, L. A. & Thorpe, M. F. Protein flexibility predictions using graph theory. *Proteins: Structure, Function, and Bioinformatics* **44**, 150-165 (2001).

7 Han, B., Liu, Y., Ginzinger, S. W. & Wishart, D. S. SHIFTX2: significantly improved protein chemical shift prediction. *Journal of biomolecular NMR* **50**, 43 (2011).

8 Borgnia, M. J. *et al.* Using cryo-EM to map small ligands on dynamic metabolic enzymes: studies with glutamate dehydrogenase. *Mol. Pharmacol.* **89**, 645-651 (2016).
